# Proceedings of the 2016 Childhood Arthritis and Rheumatology Research Alliance (CARRA) Scientific Meeting: Toronto, Canada. 14-17 April 2016

**DOI:** 10.1186/s12969-016-0098-0

**Published:** 2016-07-12

**Authors:** Lampros Fotis, Nur Shaikh, Kevin Baszis, Anthony French, Phillip Tarr, Sriharsha Grevich, Peggy Lee, Sarah Ringold, Brian Leroux, Hannah Leahey, Megan Yuasa, Jessica Foster, Jeremy Sokolove, Lauren Lahey, William Robinson, Joshua Newsom, Anne Stevens, Rie Karasawa, Mayumi Tamaki, Megumi Tanaka, Toshiko Sato, Kazuo Yudoh, James N. Jarvis, Halima Moncrieffe, Mark F. Bennett, Monica Tsoras, Lorie Luyrink, Huan Xu, Sampath Prahalad, Paula Morris, Jason Dare, Peter A. Nigrovic, Margalit Rosenkranz, Mara Becker, Kathleen M. O’Neil, Thomas Griffin, Daniel J. Lovell, Alexei A. Grom, Mario Medvedovic, Susan D. Thompson, Lisha Zhu, Kaiyu Jiang, Laiping Wong, Michael J Buck, Yanmin Chen, Halima Moncrieffe, Laura Brungs, Tao Liu, Ting Wang, James N Jarvis, Khaled Alsaeid, Jasim Alfailakawi, Hamid Alenezi, Hazim Alsaeed, Tim Beukelman, Marc Natter, Norm Ilowite, Kelly Mieszkalski, Grendel Burrell, Brian Best, Helen Bristow, Shannon Carr, Anne Dennos, Rachel Kaufmann, Yukiko Kimura, Laura Schanberg, Peter R. Blier, Alexis Boneparth, Scott E. Wenderfer, L. Nandini Moorthy, Suhas M. Radhakrishna, Anna Carmela P. Sagcal-Gironella, Emily von Scheven, Kader Cetin Gedik, Salma Siddique, Cassyanne L. Aguiar, Doruk Erkan, Ezra Cohen, Yvonne Lee, Michelle Dossett, Darshan Mehta, Roger Davis, Mileka Gilbert, Beatrice Goilav, Esra Meidan, Joyce Hsu, Alexis Boneparth, Anabelle Chua, Stacy Ardoin, Scott E. Wenderfer, Emily Von Scheven, Natasha M. Ruth, Joyce Hui-Yuen, Kader Cetin Gedik, Liza Bermudez, Ashlea Cook, Lisa Imundo, Amy Starr, Andrew Eichenfield, Anca Askanase, Ginger Janow, Laura E. Schanberg, Soko Setoguchi, Victor Hasselblad, Elizabeth D. Mellins, Rayfel Schneider, Yukiko Kimura, The CARRA Legacy Registry Investigators, Yukiko Kimura, Sriharsha Grevich, Timothy Beukelman, Esi Morgan, T. Brent Graham, Maria Ibarra, Yonit Sterba Ruas, Marisa Klein-Gitelman, Karen Onel, Sampath Prahalad, Marilynn Punaro, Sarah Ringold, Dana Toib, Heather Van Mater, Jennifer E. Weiss, Pamela F. Weiss, Kelly Mieszkalski, Laura E. Schanberg, Timothy S. H. Kwok, Jacinthe Bisaillon, Christine Smith, Lucie Brosseau, Jennifer Stinson, Adam M. Huber, Ciaran M. Duffy, Karine Toupin April, Laura B. Lewandowski, Christiaan Scott, Suzanne C. Li, Kathryn S. Torok, C. Egla Rabinovich, Sandy D. Hong, Mara L Becker, Fatma Dedeoglu, Maria F. Ibarra, Polly J Ferguson, Rob C. Fuhbrigge, Katie G. Stewart, Elena Pope, Ronald M. Laxer, Thomas G. Mason, Gloria C. Higgins, Xiaohu Li, Marilynn G. Punaro, George Tomlinson, Eleanor Pullenayegum, John Matelski, Laura Schanberg, Brian M. Feldman, Kalpana Manthiram, Hernan Correa, Kathryn Edwards, Edward J. Oberle, Michelle Bayer, Dominic O. Co, Hatice Ezgi Baris, Yvonne Chiu, Adam Huber, Susan Kim, Edward J. Oberle, Timothy Beukelman, Amir B. Orandi, Kevin W. Baszis, Vikas Dharnidharka, Mark F. Hoeltzel, for the CARRA JDM Committee, Ann Reed, Adam Huber, George Tomlinson, Eleanor Pullenayegum, John Matelski, Y. Ingrid Goh, Laura Schanberg, Brian M. Feldman, Anja Schnabel, Ursula Range, Gabriele Hahn, Timo Siepmann, Reinhard Berner, Christian Michael Hedrich, Brandi Stevens, Kathryn S. Torok, Suzanne Li, Nicole Hershey, Megan Curran, Gloria Higgins, Katharine Moore, Egla Rabinovich, Anne M. Stevens, for the CARRA Registry Investigators, Jennifer Stinson, Mark Connelly, Adam Huber, Nadia Luca, Lynn Spiegel, Argerie Tsimicalis, Stephanie Luca, Naweed Tajuddin, Roberta Berard, Julia Barsalou, Sarah Campillo, Paul Dancey, Ciaran Duffy, Brian Feldman, Nicole Johnson, Patrick McGrath, Natalie Shiff, Shirley Tse, Lori Tucker, Charles Victor, Jennifer Stinson, Chitra Lalloo, Lauren Harris, Joseph Cafazzo, Lynn Spiegel, Brian Feldman, Nadia Luca, Ronald Laxer, Danielle R. Bullock, Richard K. Vehe, Lei Zhang, Colleen K. Correll, Suhas Ganguli, Max Shenberger, Ritesh Korumilli, Beth Gottlieb, Martha Rodriguez, Deirdre de Ranieri, Karen Onel, Linda Wagner-Weiner, Melissa Tesher, Elizabeth Roth Wojcicki, Kristyn L. Maletta, Dominic O. Co, Marsha Malloy, Sarah Thomson, Judyann C. Olson, Scott E. Wenderfer, Mileka Gilbert, Joyce Hsu, Sangeeta Sule, Tamar B. Rubinstein, Beatrice Goilav, Daryl M. Okamura, Annabelle Chua, Laurence A. Greenbaum, Jerome C. Lane, Emily von Scheven, Stacy P. Ardoin, Natasha M. Ruth, Jennifer M. P. Woo, Marsha M. Malloy, James A. Jegers, Dustin J. Hahn, Mary K. Hintermeyer, Stacey M. Martinetti, Gretchen R. Heckel, Elizabeth L. Roth-Wojcicki, Dominic O. Co

**Affiliations:** Washington University School of Medicine, St Louis, Missouri USA; Department of Pediatrics, Division of Rheumatology, University of Washington, Seattle, WA USA; Department of Oral Medicine, University of Washington, Seattle, WA USA; Department of Rheumatology, Seattle Children’s Hospital, Seattle, WA USA; Department of Oral Health Sciences, University of Washington, Seattle, WA USA; University of Washington, Seattle, WA USA; Seattle Children’s Research Institute, Seattle, WA USA; Department of Medicine, Division of Immunology and Rheumatology, Stanford University, Stanford, CA USA; St. Marianna University School of Medicine, Kawasaki, Japan; University at Buffalo, State University of New York, Buffalo, NY USA; Cincinnati Children’s Hospital Medical Center, Cincinnati, OH USA; University of Cincinnati, Cincinnati, OH USA; Emory University, Atlanta, GA USA; Arkansas Children’s Hospital Research Institute, Little Rock, AR USA; Boston Children’s Hospital, Boston, MA USA; Children’s Hospital of Pittsburgh of UPMC, Pittsburgh, PA USA; Children’s Mercy Hospital, Kansas City, MO USA; Riley Hospital for Children at IU Health, Indianapolis, IN USA; Levine Children’s Hospital, Charlotte, NC USA; University at Buffalo, The State University of New York, Buffalo, NY USA; Cincinnati Children’s Medical Ctr, Cincinnati, OH USA; Washington University, St. Louis, MO USA; Department of Pediatrics, Kuwait University, Kuwait City, Kuwait; Mubarak Hospital, Jabriya, Kuwait; University of Alabama at Birmingham, Birmingham, AL USA; Tufts University, Medford, MA USA; The Children’s Hospital at Montefiore, Bronx, NY USA; CARRA, Inc, Durham, NC USA; Duke University, Durham, NC USA; Hackensack University Medical Center, Hackensack, NJ USA; Harvard University, Boston, MA USA; Baystate Children’s Hospital, Springfield, MA USA; Tufts University School of Medicine, Boston, MA USA; Rutgers-Robert Wood Johnson Medical School, New Brunswick, NJ USA; Baylor College of Medicine, Houston, TX USA; Rady Children’s Hospital, San Diego, CA USA; University of California at San Francisco, San Francisco, CA USA; Cohen Children’s Medical Center of New York, New York, USA; Hospital for Special Surgery-Weill Cornell Medical Center, New York, NY USA; Boston Children’s Hospital, Boston, MA USA; Brigham and Women’s Hospital, Boston, MA USA; Massachusetts General Hospital, Boston, MA USA; Beth Israel Deaconess, Boston, MA USA; Medical University of South Carolina, Charleston, SC USA; Children’s Hospital at Montefiore, Bronx, NY USA; Boston Children’s Hospital, Boston, MA USA; Stanford University, Stanford, CA USA; Rutgers University, Rutgers, NJ USA; Duke University, Durham, NC USA; Nationwide Children’s Hospital, Columbus, OH USA; Baylor College of Medicine, Houston, TX USA; UCSF, San Francisco, CA USA; Division of Pediatric Rheumatology, Cohen Children’s Medical Center, New Hyde Park, NY USA; Division of Pediatric Rheumatology, Morgan Stanley Children’s Hospital of New York-Presbyterian, Columbia University Medical Center, New York, NY USA; Division of Adult Rheumatology, Columbia University Medical Center, New York, NY USA; Pediatrics, Joseph M Sanzari Children’s Hospital, Hackensack, NJ USA; Pediatrics, Duke University, Durham, NC USA; Duke Clinical Research Institute, Durham, NC USA; Pediatrics, Stanford University, Stanford, USA; Pediatrics, Hospital for Sick Children and University of Toronto, Toronto, Canada; Hackensack University Medical Center, Hackensack, NJ USA; Seattle Children’s Hospital, Seattle, WA USA; University of Alabama, Tuscaloosa, AL USA; Cincinnati Children’s Hospital Medical Center, Cincinnati, OH USA; Vanderbilt University, Vanderbilt, TN USA; Children’s Mercy Hospital, Kansas City, MO USA; Children’s Hospital of Montefiore, Bronx, NY USA; Lurie Children’s Hospital of Chicago, Chicago, IL USA; Comer Children’s Hospital of Chicago, Chicago, IL USA; Emory Children’s Hospital, Atlanta, GA USA; Texas Scottish Rite Hospital, Dallas, TX USA; St. Christopher’s Hospital for Children, Philadelphia, PA USA; Duke University, Durham, NC USA; Children’s Hospital of Philadelphia, Philadelphia, PA USA; CARRA, Durham, NC USA; Undergraduate Medical Education, Faculty of Medicine, University of Ottawa, Ottawa, Ontario Canada; School of Nursing Sciences, Faculty of Health Sciences, University of Ottawa, Ottawa, Ontario Canada; School of Epidemiology, Public Health and Preventive Medicine, Faculty of Medicine, University of Ottawa, Ottawa, Ontario Canada; School of Rehabilitation Sciences, Faculty of Health Sciences, University of Ottawa, Ottawa, Ontario Canada; Hospital for Sick Children, Lawrence S. Bloomberg Faculty of Nursing University of Toronto, Toronto, Ontario Canada; Department of Pediatrics, IWK Health Centre, Dalhousie University, Halifax, Nova Scotia Canada; Department of Pediatrics, Faculty of Medicine, University of Ottawa, Children’s Hospital of Eastern Ontario Research Institute, Ottawa, Ontario Canada; Department of Pediatrics, Children’s Hospital of Eastern Ontario Research Institute, Faculty of Medicine, School of Rehabilitation Sciences, Faculty of Health Sciences, University of Ottawa, Ottawa, Ontario Canada; Pediatric Rheumatology, Duke University Medical Center, Durham, NC USA; Duke Global Health Institute, Duke University, Durham, NC USA; Paediatric Rheumatology, Red Cross War Memorial Children’s Hospital and University of Cape Town, Cape Town, South Africa; National Institute of Arthritis, Musculoskeletal, and Skin Diseases, NIH, Bethesda, MD USA; Hackensack University Medical Center, Hackensack, NJ USA; Children’s Hospital of Pittsburgh, UPMC, Pittsburgh, PA USA; Duke University, Durham, NC USA; University of Iowa, Iowa City, IA USA; Children’s Mercy Hospital, Kansas City, MO USA; Boston Children’s Hospital, Boston, MA USA; Texas Scottish Rite Hospital, Dallas, TX USA; Hospital for Sick Kids, Toronto, Canada; Mayo Clinic, Rochester, MN USA; Nationwide Children’s Hospital, Columbus, OH USA; Stevens Institute of Technology, Hoboken, NJ USA; Division of Pediatric Infectious Diseases, Vanderbilt University School of Medicine, Nashville, TN USA; Department of Pathology, Immunology, and Microbiology, Vanderbilt University School of Medicine, Nashville, TN USA; Nationwide Children’s Hospital, The Ohio State University, Columbus, OH USA; Children’s Hospital of Wisconsin, Medical College of Wisconsin, Milwaukee, WI USA; Boston Children’s Hospital, Boston, MA USA; IWK Health Centre, Dalhousie University, Halifax, NS Canada; Harvard Medical School, Boston, MA USA; Nationwide Children’s Hospital, Columbus, OH USA; The Ohio State University, Columbus, OH USA; University of Alabama at Birmingham, Birmingham, AL USA; St. Louis Children’s Hospital, Washington University School of Medicine, St. Louis, MO USA; Mott Children’s Hospital, University of Michigan Medical School, Ann Arbor, MI USA; Duke University, Durham, NC USA; Dalhousie University, Halifax, NS Canada; University of Toronto, Toronto, Canada; Pediatric Rheumatology and Immunology, Children’s Hospital Dresden, Universitätsklinikum Carl Gustav Carus, Technische Universität Dresden, Dresden, Germany; Institute for Medical Informatics and Biometry, Universitätsklinikum Carl Gustav Carus, Technische Universität Dresden, Dresden, Germany; Department of Radiology, Universitätsklinikum Carl Gustav Carus, Technische Universität Dresden, Dresden, Germany; Division of Health Care Sciences, Center for Clinical Research and Management Education, Dresden International University, Dresden, Germany; Children’s Hospital of Pittsburgh of UPMC, Pittsburgh, PA USA; Hackensack University Medical Center, Hackensack, NJ USA; Northwestern University Feinberg School of Medicine, Chicago, IL USA; Nationwide Children’s Hospital, Columbus, OH USA; Children’s Hospital of Denver, Denver, CO USA; Duke University Medical Center, Durham, NC USA; Seattle Children’s Research Institute, University of Washington, Seattle, WA USA; Childhood Arthritis and Rheumatology Research Alliance, ᅟ, USA; The Hospital for Sick Children, University of Toronto, Toronto, Canada; IWK Health Centre, Halifax, Nova Scotia Canada; McGill University, Montreal, Canada; Children’s Hospital of Western Ontario, London, Canada; CHU Ste-Justine, Montreal, Quebec, Canada; Montreal Children’s Hospital, Montreal, Canada; Memorial University of Newfoundland, Newfoundland, Canada; Children’s Hospital of Eastern Ontario, Ottawa, Canada; Alberta Children’s Hospital, Calgary, Alberta Canada; University of Florida, Gainesville, FL USA; British Columbia Children’s Hospital, Vancouver, British Columbia Canada; University of Toronto, Toronto, Canada; University of Kansas Medical Center, Kansas City, MO USA; The Hospital for Sick Children, Toronto, Canada; University of Toronto, Toronto, Canada; University Health Network, Toronto, Canada; Centre for Global eHealth Innovation, Toronto, Canada; Alberta Children’s Hospital, Calgary, Alberta Canada; Division of Pediatric Rheumatology, Department of Pediatrics, University of Minnesota Masonic Children’s Hospital, Minneapolis, MN USA; Clinical and Translational Sciences Institute, University of Minnesota, Minneapolis, MN USA; Pediatric Rheumatology, Cohen Children’s Medical Center, New York, NY 11040 USA; Pediatrics, Flushing Hospital Medical Center, New York, NY 11355 USA; University of Chicago Medicine Comer Children’s Hospital, Chicago, IL USA; Medical College of WI, Milwaukee, WI USA; National Outcomes Center, Milwaukee, WI USA; Children’s Hospital of Wisconsin, Milwaukee, WI USA; Baylor College of Medicine, Houston, TX USA; Medical University of South Carolina, Charleston, SC USA; Stanford University, Stanford, CA USA; Johns Hopkins Children’s Hospital, Baltimore, MD USA; Children’s Hospital at Montefiore, Bronx, NY USA; Seattle Children’s Hospital, Seattle, WA USA; Duke University, Durham, NC USA; Emory University, Atlanta, GA USA; Northwestern University, Evanston, IL USA; University of California San Francisco, San Francisco, CA USA; Nationwide Children’s Hospital, Columbus, OH USA; University of Wisconsin-Milwaukee, Milwaukee, WI USA; Medical College of Wisconsin, Milwaukee, WI USA; Children’s Hospital of Wisconsin, Milwaukee, WI USA; Parent of Patient, Milwaukee, WI USA

## Abstract

P1 Serologic evidence of gut-driven systemic inflammation in juvenile idiopathic arthritis

Lampros Fotis, Nur Shaikh, Kevin Baszis, Anthony French, Phillip Tarr

P2 Oral health and anti-citrullinated peptide antibodies (ACPA) in juvenile idiopathic arthritis

Sriharsha Grevich, Peggy Lee, Sarah Ringold, Brian Leroux, Hannah Leahey, Megan Yuasa, Jessica Foster, Jeremy Sokolove, Lauren Lahey, William Robinson, Joshua Newsom, Anne Stevens

P3 Novel autoantigens for endothelial cell antibodies in pediatric rheumatic diseases identified by proteomics

Rie Karasawa, Mayumi Tamaki, Megumi Tanaka, Toshiko Sato, Kazuo Yudoh, James N. Jarvis

P4 Transcriptional profiling reveals monocyte signature associated with JIA patient poor response to methotrexate

Halima Moncrieffe, Mark F. Bennett, Monica Tsoras, Lorie Luyrink, Huan Xu, Sampath Prahalad, Paula Morris, Jason Dare, Peter A. Nigrovic, Margalit Rosenkranz, Mara Becker, Kathleen M. O’Neil, Thomas Griffin, Daniel J. Lovell, Alexei A. Grom, Mario Medvedovic, Susan D. Thompson

P5 A multi-dimensional genomic map for polyarticular juvenile idiopathic arthritis

Lisha Zhu, Kaiyu Jiang, Laiping Wong, Michael J Buck, Yanmin Chen, Halima Moncrieffe, Laura Brungs, Tao Liu, Ting Wang, James N Jarvis

P6 Tocilizumab for treatment of children with refractory JIA

Khaled Alsaeid, Jasim Alfailakawi, Hamid Alenezi, Hazim Alsaeed

P7 Clinical characteristics of the initial patients enrolled in the Childhood Arthritis and Rheumatology Research Alliance (CARRA) Registry

Tim Beukelman, Marc Natter, Norm Ilowite, Kelly Mieszkalski, Grendel Burrell, Brian Best, Helen Bristow, Shannon Carr, Anne Dennos, Rachel Kaufmann, Yukiko Kimura, Laura Schanberg

P8 Comparative performance of small and large clinical centers in a comprehensive pediatric rheumatology disease registry

Peter R Blier

P9 Clinical characteristics of children with membranous lupus nephritis: The Childhood Arthritis and Rheumatology Research Alliance Legacy Registry

Alexis Boneparth, Scott E. Wenderfer, L. Nandini Moorthy, Suhas M. Radhakrishna, Anna Carmela P. Sagcal-Gironella, Emily von Scheven

P10 Rituximab use in pediatric lupus anticoagulant hypoprothrombinemia syndrome - a two center experience

Kader Cetin Gedik, Salma Siddique, Cassyanne L. Aguiar, Doruk Erkan

P11 Predictors of complementary and alternative medicine use and response in children with musculoskeletal conditions

Ezra Cohen, Yvonne Lee, Michelle Dossett, Darshan Mehta, Roger Davis

P12 Comparison of pediatric rheumatology and nephrology survey results for the treatment of refractory proliferative lupus nephritis and renal flare in juvenile SLE

Mileka Gilbert, Beatrice Goilav, Esra Meidan, Joyce Hsu, Alexis Boneparth, Anabelle Chua, Stacy Ardoin, Scott E. Wenderfer, Emily Von Scheven, Natasha M. Ruth

P13 Transitioning lupus patients from pediatric to adult rheumatology

Joyce Hui-Yuen, Kader Cetin Gedik, Liza Bermudez, Ashlea Cook, Lisa Imundo, Amy Starr, Andrew Eichenfield, Anca Askanase

P14 The systemic juvenile idiopathic arthritis cohort of the Childhood Arthritis & Rheumatology Research Alliance Registry

Ginger Janow, Laura E. Schanberg, Soko Setoguchi, Victor Hasselblad, Elizabeth D. Mellins, Rayfel Schneider, Yukiko Kimura, The CARRA Legacy Registry Investigators

P15 Results of the pilot study of the Childhood Arthritis and Rheumatology Research Alliance (CARRA) consensus treatment plans for new-onset systemic juvenile idiopathic arthritis

Yukiko Kimura, Sriharsha Grevich, Timothy Beukelman, Esi Morgan, T Brent Graham, Maria Ibarra, Yonit Sterba Ruas, Marisa Klein-Gitelman, Karen Onel, Sampath Prahalad, Marilynn Punaro, Sarah Ringold, Dana Toib, Heather Van Mater, Jennifer E. Weiss, Pamela F. Weiss, Kelly Mieszkalski, Laura E. Schanberg

P16 A systemic review of pain relief modalities in juvenile idiopathic arthritis: First step in developing a novel decision support intervention

Timothy S. H. Kwok, Jacinthe Bisaillon, Christine Smith, Lucie Brosseau, Jennifer Stinson, Adam M. Huber, Ciaran M. Duffy, Karine Toupin April

P17 Barriers and facilitators to care retention for pediatric systemic lupus erythematous patients in South Africa: A qualitative study

Laura B Lewandowski, Christiaan Scott

P18 Evaluating the feasibility of conducting comparative effectiveness studies in juvenile Localized Scleroderma (jLS)

Suzanne C. Li, Kathryn S. Torok, C. Egla Rabinovich, Sandy D. Hong, Mara L Becker, Fatma Dedeoglu, Maria F. Ibarra, Polly J Ferguson, Rob C. Fuhbrigge, Katie G. Stewart, Elena Pope, Ronald M. Laxer, Thomas G. Mason, Gloria C. Higgins, Xiaohu Li, Marilynn G. Punaro, George Tomlinson, Eleanor Pullenayegum, John Matelski, Laura Schanberg, Brian M. Feldman

P19 Tonsillar histology in patients with periodic fever, aphthous stomatitis, pharyngitis, adenitis (PFAPA) syndrome

Kalpana Manthiram, Hernan Correa, Kathryn Edwards

P20 Clinical course of juvenile dermatomyositis presenting as skin predominant disease

Edward J. Oberle, Michelle Bayer, Dominic O. Co, Hatice Ezgi Baris, Yvonne Chiu, Adam Huber, Susan Kim

P21 A Survey of musculoskeletal ultrasound practices of pediatric rheumatologists in North America

Edward J Oberle, Timothy Beukelman

P22 Assessment, classification and treatment of calcinosis as a complication of juvenile dermatomyositis: A survey of pediatric rheumatologists by the Childhood Arthritis and Rheumatology Research Alliance

Amir B. Orandi, Kevin W. Baszis, Vikas Dharnidharka, Mark F. Hoeltzel, for the CARRA JDM Committee

P23 CARRA dermatomyositis CTP pilot study

Ann Reed, Adam Huber, George Tomlinson, Eleanor Pullenayegum, John Matelski, Y. Ingrid Goh, Laura Schanberg, Brian M. Feldman

P24 Unexpectedly high incidences and prolonged disease activity in children with chronic non-bacterial osteomyelitis (CNO) as compared to bacterial osteomyelitis

Anja Schnabel, Ursula Range, Gabriele Hahn, Timo Siepmann, Reinhard Berner, Christian Michael Hedrich

P25 Juvenile systemic sclerosis cohort within the Childhood Arthritis and Rheumatology Research Alliance (CARRA) Legacy Registry: Follow up characteristics

Brandi Stevens, Kathryn S. Torok, Suzanne Li, Nicole Hershey, Megan Curran, Gloria Higgins, Katharine Moore, Egla Rabinovich, Anne M. Stevens, for the CARRA Registry Investigators

P26 Development and usability testing of an iPad and desktop psycho-educational game for children with Juvenile Idiopathic Arthritis and their parents

Jennifer Stinson, Mark Connelly, Adam Huber, Nadia Luca, Lynn Spiegel, Argerie Tsimicalis, Stephanie Luca, Naweed Tajuddin, Roberta Berard, Julia Barsalou, Sarah Campillo, Paul Dancey, Ciaran Duffy, Brian Feldman, Nicole Johnson, Patrick McGrath, Natalie Shiff, Shirley Tse, Lori Tucker, Charles Victor

P27 *iCanCope*^TM^: User-centred design and development of a smartphone app to support self-management for youth with arthritis pain

Jennifer Stinson, Chitra Lalloo, Lauren Harris, Joseph Cafazzo, Lynn Spiegel, Brian Feldman, Nadia Luca, Ronald Laxer

P28 Accessing pediatric rheumatology care: Despite barriers, few parents prefer telemedicine

Danielle R. Bullock, Richard K. Vehe, Lei Zhang, Colleen K. Correll^1^

P29 Exploration of factors contributing to time to achieve clinically inactive disease (CID) in juvenile idiopathic arthritis (JIA): A preliminary report

Suhas Ganguli, Max Shenberger, Ritesh Korumilli, Beth Gottlieb

P30 Pediatric rheumatology referral patterns: Presenting complaints of new patients at a large, urban academic center

Martha Rodriguez, Deirdre de Ranieri, Karen Onel, Linda Wagner-Weiner, Melissa Tesher

P31 Quality improvement (QI) initiatives in childhood systemic lupus erythematosus (cSLE)

Elizabeth Roth Wojcicki, Kristyn L. Maletta, Dominic O. Co, Marsha Malloy, Sarah Thomson, Judyann C. Olson

P32 Proliferative lupus nephritis in juvenile SLE: Support from the pediatric nephrology community for the definitions of responsiveness and flare in the 2012 consensus treatment plans

Scott E. Wenderfer, Mileka Gilbert, Joyce Hsu, Sangeeta Sule, Tamar B. Rubinstein, Beatrice Goilav, Daryl M. Okamura, Annabelle Chua, Laurence A. Greenbaum, Jerome C. Lane, Emily von Scheven, Stacy P. Ardoin, Natasha M. Ruth

P33 The steroid taper app: Making of a mobile app

Jennifer M. P. Woo, Marsha M. Malloy, James A. Jegers, Dustin J. Hahn, Mary K. Hintermeyer, Stacey M. Martinetti, Gretchen R. Heckel, Elizabeth L. Roth-Wojcicki, Dominic O. Co

## P1 Serologic evidence of gut-driven systemic inflammation in juvenile idiopathic arthritis

### Lampros Fotis, Nur Shaikh, Kevin Baszis, Anthony French, Phillip Tarr

#### Washington University School of Medicine, St Louis, Missouri, USA

##### **Correspondence:** Lampros Fotis (fotis_l@kids.wustl.edu) – Washington University School of Medicine, St Louis, Missouri, USA

**Background**

Evolving data link juvenile idiopathic arthritis (JIA) to environmental factors and gut microbes, which may trigger the innate immune system through bacterial lipopolysaccharides. We hypothesized that children with new onset JIA have increased intestinal bacterial translocation and endotoxinemia.

**Methods**

65 new onset, systemic treatment naive JIA patients (polyarticular JIA, n = 21, oligoarticular JIA, n = 29 and spondylarthropathies, n = 15) and 14 healthy controls participated in the study. We determined the plasma immunoglobulin reactivity against *Escherichia coli* lipopolysaccharide (LPS). We measured the levels of lipopolysaccharide binding protein (LBP), and alpha-1-acid glycoprotein (A1AGP), which are acute phase proteins related to intestinal bacterial translocation, and C-reactive protein (CRP). Assays were performed using commercial EIA kits (Hycult, R&D systems) or laboratory-prepared LPS.

**Results**

Children with polyarticular JIA have statistically significant increased concentrations of circulating anti-LPS antibodies, LBP, CRP, and A1AGP compared to healthy controls (p < 0.05). The concentration of LBP, CRP and A1AGP in patients with polyarticular JIA significantly differs from the oligoarticular JIA group (p < 0.05), but not spondylarthropathies. Spondylarthropathy patients have increased circulating anti-LPS antibodies compared to healthy controls (p < 0.05). There are no statistically significant differences between oligoarticular JIA patients and healthy controls. LBP and A1AGP levels correlate with CRP levels (r = 0.787 and r = 0.635 respectively).

**Conclusions**

Children with polyarticular JIA and spondylarthropathies have evidence of increased exposure to gut bacteria, as reflected by anti-LPS antibodies and LBP. The degree of exposure is proportional to the degree of systemic inflammation. These data imply that the intestine maybe the source of stimulation for the immune system in JIA.

## P2 Oral health and anti-citrullinated peptide antibodies (ACPA) in juvenile idiopathic arthritis

### Sriharsha Grevich^1^, Peggy Lee^2^, Sarah Ringold^3^, Brian Leroux^4^, Hannah Leahey^5^, Megan Yuasa^5^, Jessica Foster^6^, Jeremy Sokolove^7^, Lauren Lahey^7^, William Robinson^7^, Joshua Newsom^5^, Anne Stevens^1,6^

#### ^1^Department of Pediatrics, Division of Rheumatology, University of Washington, Seattle, WA, USA; ^2^Department of Oral Medicine, University of Washington, Seattle, WA, USA; ^3^Department of Rheumatology, Seattle Children's Hospital, Seattle, WA, USA; ^4^Department of Oral Health Sciences, University of Washington, Seattle, WA, USA; ^5^University of Washington, Seattle, WA, USA; ^6^Seattle Children's Research Institute, Seattle, WA, USA; ^7^Department of Medicine, Division of Immunology and Rheumatology, Stanford University, Stanford, CA, USA

##### **Correspondence:** Sriharsha Grevich (sriharsha.grevich@seattlechildrens.org) – Department of Pediatrics, Division of Rheumatology, University of Washington, Seattle, WA, USA

**Background**

Oral pathogens that cause periodontitis have been implicated as triggers for rheumatoid arthritis (RA), with antibodies to bacterial citrullinated proteins (ACPA) suggested as a potential mechanism for the transfer of oral inflammation to the joints. Although periodontitis is rare in children, gingivitis, present in 50 % of adolescents, is a chronic inflammatory precursor to periodontitis. Patients with juvenile idiopathic arthritis (JIA) rarely produce ACPA, but patients with polyarticular JIA who carry the ACPA, are considered to have more aggressive arthritis, similar to adult RA patients. Because adult RA is associated with gingival inflammation and periodontitis, we tested for an association between ACPA and dental clinical outcomes in JIA. Our study objectives were to test for increased frequency of gingivitis in children with JIA compared to controls, and to test for correlations between ACPA and dental clinical outcomes in JIA patients and dental controls.

**Methods**

This was a cross-sectional study of 85 patients with JIA, 62 dental patient controls and 11 healthy patient controls at the Seattle Children’s Hospital (SCH) Rheumatology and Dental Clinics. Serum from an additional historic cohort of 30 healthy children was used to study ACPA. Rheumatologists and dentists completed the oral and joint exams blinded to each other’s findings. ACPA were detected with a commercial CCP3 assay and also to 29 citrullinated full length proteins or peptides within these proteins using a custom Bio-Plex bead array. For each peptide, elevated levels were defined to be values larger than the maximum value observed for the healthy controls. Patients were defined to have elevated ACPA if they had elevated levels for more than 4 of the proteins. The prevalence of elevated ACPA was compared across groups using the chi-squared test.

**Results**

Although JIA patients overall had better oral health than controls (lower indices for plaque, caries and gingivitis), they had significantly more bleeding on probing of the gingiva, the most specific sign of active inflammation (percentage of sites bleeding 17 % vs 8 %, p = 0.007). There was no correlation between JIA disease activity or immunosuppression with dental indices. ACPA were detected in only three JIA patients by commercial CCP3 assay, but elevated levels were detected by the custom array in 15/41 (37 %) of poly JIA, 14/36 (39 %) of oligo JIA, and 19/57 (33 %) of dental controls. Antibodies to more than 4 citrullinated peptides were most common in poly JIA 9/41 (22 %) compared with 1/36 (3 %) for oligo JIA, and 2/57 (4 %) for dental controls, (p = 0.002 by chi-squared test). We found no associations between ACPA status and any dental index. **Conclusions**

Gingival inflammation was associated with JIA, suggesting a systemic immune response could be triggered by oral bacteria involved in both oral and synovial inflammation. More sensitive and specific tests for ACPA may lead to improved prognosis and understanding of the triggers for arthritis in children.

## P3 Novel autoantigens for endothelial cell antibodies in pediatric rheumatic diseases identified by proteomics

### Rie Karasawa^1^, Mayumi Tamaki^1^, Megumi Tanaka^1^, Toshiko Sato^1^, Kazuo Yudoh^1^, James N. Jarvis^2^

#### ^1^St. Marianna University School of Medicine, Kawasaki, Japan; ^2^University at Buffalo, State University of New York, Buffalo, NY, USA

##### **Correspondence:** Rie Karasawa (r2karasawa@marianna-u.ac.jp) – St. Marianna University School of Medicine, Kawasaki, Japan

**Background**

Juvenile dermatomyositis (JDM) is recognized as a systemic autoimmune vasculopathy. While more rare than and juvenile idiopathic arthritis (JIA), JDM shares common features with JIA (e.g., many JDM patients have arthritis) and each is representative of the spectrum of rheumatic disease manifestations in children. However the pathogenesis of these diseases remains to be fully solved. More research is needed to identify disease-specific biomarkers for diagnosis and monitoring of pediatric rheumatic diseases. Anti-endothelial cell antibodies (AECA) have been detected in rheumatic diseases such as Kawasaki disease, however, roles and functions of AECA are still uncertain. Multiple studies have reported a relationship between inflammation and endothelial cell functions (Nat Rev Immunol. 2007; 7:803). We aimed to detect target antigens for AECA in patients with pediatric rheumatic diseases comprehensively by proteomics and investigated clinical and diagnostic significance of them.

**Methods**

To detect target antigens on endothelial cells for AECA, we separated proteins extracted from human aortic endothelial cells (HAEC) by two-dimensional electrophoresis (2DE) and then transferred them onto membranes. Antigens that were positive only in pediatric rheumatic disease sera from three patients with JDM and two patients with JIA but not in healthy donor sera were detected by western blotting (WB). The detected proteins were identified using peptide mass finger-printing. Bound IgG antibodies to HAEC antigens were detected using standard methods.

**Results**

We detected eighteen protein spots as pediatric rheumatic disease-specific autoantigens by 2DE-WB. We successfully identified 738 proteins out of these spots that were candidate targets of autoantibodies in pediatric rheumatic diseases. Antibodies appeared to target proteins with specific functions, e.g., ATP-related proteins (37 %), muscle-related proteins (19 %), calcium regulated protein and/or calcium binding proteins (13 %) and redox related proteins (10 %). Furthermore, half of the identified target antigens represented membrane proteins. Among the 738 candidate HAEC autoantigens were myosin light polypeptide 6 (MYL6) and myosin-9 (MYH9). IgG autoantibodies to MYL6 were detected in 20 % of the patients with JDM (n = 61) using ELISA assays. However, 50 % of the untreated JDM patients with active disease (n = 10) had anti-MYL6 antibodies, in contrast to 12 % (p < 0.05) of the patients with JIA (n = 17) and in 16 % (p = 0.05) of control children (n = 25) tested on the same ELISA assays. IgG autoantibodies to MYH9 were detected in 31 % of the active patients with JDM (n = 35), (50 % of the untreated JDM patients with active disease), in comparison to 27 % of the patients with JIA (n = 15) and 12 % (p < 0.05) of control children.

**Conclusions**

IgG antibodies to MYL6 and MYH9 were detected in the sera from patients with pediatric rheumatic diseases, respectively. These antibodies might be useful markers for pediatric rheumatic diseases. Proteomic approaches are a powerful tool for identifying target antigens for AECA.

## P4 Transcriptional profiling reveals monocyte signature associated with JIA patient poor response to methotrexate

### Halima Moncrieffe^1,2^, Mark F. Bennett^2^, Monica Tsoras^1^, Lorie Luyrink^1^, Huan Xu^2^, Sampath Prahalad^3^, Paula Morris^4^, Jason Dare^4^, Peter A. Nigrovic^5^, Margalit Rosenkranz^6^, Mara Becker^7^, Kathleen M. O’Neil^8^, Thomas Griffin^9^, Daniel J. Lovell^1^, Alexei A. Grom^1^, Mario Medvedovic^2^, Susan D. Thompson^1,2^

#### ^1^Cincinnati Children’s Hospital Medical Center, Cincinnati, OH, USA; ^2^University of Cincinnati, Cincinnati, OH, USA; ^3^Emory University, Atlanta, GA, USA; ^4^Arkansas Children’s Hospital Research Institute, Little Rock, AR, USA; ^5^Boston Children’s Hospital, Boston, MA, USA; ^6^Children’s Hospital of Pittsburgh of UPMC, Pittsburgh, PA, USA; ^7^Children’s Mercy Hospital, Kansas City, MO, USA; ^8^Riley Hospital for Children at IU Health, Indianapolis, IN, USA; ^9^Levine Children’s Hospital, Charlotte, NC, USA

##### **Correspondence:** Halima Moncrieffe (halima.moncrieffe@cchmc.org) – Cincinnati Children’s Hospital Medical Center, Cincinnati, OH, USA

Halima Moncrieffe and Mark F. Bennett are joint first authors.

**Background**

Response to methotrexate (MTX), one of the main treatments for juvenile idiopathic arthritis (JIA), is highly variable in these patients. The mechanisms that lead to poor MTX response remain ill-defined. The aim of this study was to identify a gene expression transcriptional signature associated with poor response to MTX in children with JIA.

**Methods**

RNA extracted from peripheral blood mononuclear cells (PBMC) collected from 47 patients with JIA prior to MTX treatment and 14 age-matched controls was sequenced. Unsupervised clustering and pathway analysis was then performed. Biological differences between all JIA patients and controls were explored by constructing a signature of differentially expressed genes. Transcriptional profiles were compared to a reference gene expression database representing sorted cell populations, including B, T and monocytes.

**Results**

A signature of 99 differentially expressed genes (Bonferroni-corrected p < 0.05) capturing the biological differences between all JIA patients and controls was identified. Unsupervised clustering of samples based on this list of 99 genes identified subgroups enriched for MTX response status. Comparing the gene signature to reference signatures from sorted cell populations revealed high concordance between the expression profile of monocytes and that of MTX non-responders. CXCL8 (IL-8) was significantly upregulated in JIA patients compared to controls (Bonferroni-corrected p = 4.12 × 10^-10^). A direct search for genes differentially expressed between all MTX responders and all MTX non-responders identified six genes (ADAMTS2, FOSL1, F3, NR4A3, NFKBID, CD83) with Bonferroni-adjusted p-values <0.05. However, this gene signature did not predict overall response status.

**Conclusions**

The variability in clinical response to methotrexate in JIA patients is associated with differences in gene transcripts modulated in monocytes. Pre-treatment PBMC gene expression profiles may serve as useful biomarkers predictive of methotrexate responsiveness.

## P5 A multi-dimensional genomic map for polyarticular juvenile idiopathic arthritis

### Lisha Zhu^1^, Kaiyu Jiang^1^, Laiping Wong^1^, Michael J Buck^1^, Yanmin Chen^1^, Halima Moncrieffe^2^, Laura Brungs^3^, Tao Liu^1^, Ting Wang^3^, James N Jarvis^1^

#### ^1^University at Buffalo, The State University of New York, Buffalo, NY, USA; ^2^Cincinnati Children's Medical Ctr, Cincinnati, OH, USA; ^3^Washington University, St. Louis, MO, USA

##### **Correspondence:** James N Jarvis (jamesjar@buffalo.edu) – University at Buffalo, The State University of New York, Buffalo, NY, USA

**Background**

Polyarticular juvenile idiopathic arthritis is a complex trait characterized by gene-environment interactions. While we are beginning to identify multiple genomic regions associated with disease risk in JIA, most of that risk is located within non-coding regions of the genome. Thus, to develop a mechanistic understanding of how genetic variance contributes to JIA, we require a detailed understanding of the non-coding genome and the functional elements located within these regions.

**Methods**

We created a multidimensional genomic map for juvenile idiopathic arthritis (JIA). Using genome-wide methylation sequencing, whole genome sequencing on the Illumina 10x platform, RNA sequencing, and chromatin immunoprecipitation-sequencing for informative histone marks (H3K4me1 and H3K27ac), we provide new insights into the interaction between genetic variation, the epigenome, and gene expression in the context of a common human disease.

**Results**

The epigenomes of JIA neutrophils display numerous differences from those from healthy children. DNA methylation changes, however, had only a weak effect on gene expression. In contrast, H3K4me and H3K27ac marks, commonly associated with enhancer functions, strongly correlated with gene expression. Furthermore, although some unique/novel enhancer marks were associated with insertion-deletion events (indels) identified on whole genome sequencing, genetic variation could account for no more than 30 % of the JIA-specific epigenome. Treatment with methotrexate was associated with a re-ordering of transcription associated with changes in both methylation and histone marks, including histone and methylation marks located within indels. This finding demonstrates the plasticity of the epigenome in this setting. Alterations in histone marks most strongly associated with the transcriptional changes that accompanied the initiation of therapy.

**Conclusions**

JIA represents a complex genetic-epigenetic trait characterized by aberrant transcription in pathologically relevant cells. Initiation of effective therapy is associated with significant re-ordering of the epigenome, even in genomic regions where there is underlying genetic variance.

## P6 Tocilizumab for treatment of children with refractory JIA

### Khaled Alsaeid^1,2^, Jasim Alfailakawi^2^, Hamid Alenezi^2^, Hazim Alsaeed^2^

#### ^1^Department of Pediatrics, Kuwait University, Kuwait City, Kuwait; ^2^Mubarak Hospital, Jabriya, Kuwait

##### **Correspondence:** Khaled Alsaeid (khaled@hsc.edu.kw) – Department of Pediatrics, Kuwait University, Kuwait City, Kuwait

**Background**

Despite the advances in treating children with juvenile idiopathic arthritis (JIA), still a proportion of such patients may be resistant to treatment with the current modalities including methotrexate and biologics. It is possible that the lack of response to treatment in some patients is due to a difference in the underlying pathophysiology, i.e. the driving cytokine rather than the actual severity of the disease.

**Methods**

The records of 16 children with different forms of JIA, all of whom were resistant to treatment with methotrexate, prednisolone, Anti-TNF and anti-IL1 were reviewed in order to analyze the response they had after treatment with tocilizumab over a period of 24 months. All patients received tocilizumab intravenous infusions according to a standard protocol, bi-weekly for patients with systemic JIA and monthly for polyarticular JIA. The following clinical and laboratory response parameters were recorded at regular interval: number of joint with active arthritis, number of joints with limited range of motion, parent global assessment of disease activity, physician global assessment of disease activity, functional class, hemoglobin level, erythrocyte sedimentation rate (ESR), C reactive protein (CRP).

**Results**

The mean age of this group of patients was 14 years, the mean age at diagnosis was 8 years, and the mean duration of disease before initiation of tocilizumab treatment was 7 months. 54 % of the children had systemic JIA while the 46 % remaining had polyarticular JIA. Of the polyarticular JIA patients 43 % were rheumatoid Factor (RF) positive. 94 % of the patients had received prior methotrexate, 75 % steroids and 88 % etanercept. The remainder were ankinra 19 %, Adalimumab 13 %, rituximab 13 % and infliximab 6 %. The mean ESR and CRP showed a dramatic drop in the first 3 months (from 60 mm/hr to less than 20, and from 9.2 to less than 0.8 respectively). The mean hemoglobin rose from 11.8 gm/l to 13.8 gm/l. The mean physician and parent global assessment of disease activity showed significant improvement. Lastly, the mean number of joints with active arthritis and joints with limited range of motion fell from 8 and 4 respectively to two or less joints at 3 months after starting treatment. No serious adverse events were noted, only one patient had grade 2 neutropenia while 2 children had a mild increase in total cholesterol level. No cases of severe or unusual infections were reported during the 24 month period.

**Conclusions**

The dramatic response to tocilizumab in this group of patients with severe JIA resistant to conventional DMARDs, anti-TNF or anti-IL1 therapy may indicate that the disease process in such patients may not be refractory to therapy but in fact is driven by a different cytokine, in this case IL6. We conclude that when faced with a JIA patient with apparent resistance to treatment switching between different classes of biologics may produce a better outcome.

## P7 Clinical characteristics of the initial patients enrolled in the Childhood Arthritis and Rheumatology Research Alliance (CARRA) Registry

### Tim Beukelman^1^, Marc Natter^2,7^, Norm Ilowite^3^, Kelly Mieszkalski^4^, Grendel Burrell^4^, Brian Best^4^, Helen Bristow^5^, Shannon Carr^5^, Anne Dennos^5^, Rachel Kaufmann^5^, Yukiko Kimura^6^, Laura Schanberg^5^

#### ^1^University of Alabama at Birmingham, Birmingham, AL, USA; ^2^Tufts University, Medford, MA, USA; ^3^The Children's Hospital at Montefiore, Bronx, NY, USA; ^4^CARRA, Inc., Durham, NC, USA; ^5^Duke University, Durham, NC, USA; ^6^Hackensack University Medical Center, Hackensack, NJ, USA; ^7^Harvard University, Boston, MA, USA

##### **Correspondence:** Tim Beukelman (tbeukelman@peds.uab.edu) – University of Alabama at Birmingham, Birmingham, AL, USA

**Background**

In July 2015, the CARRA Registry was re-established as a multi-center observational study that collects essential data from persons with pediatric onset rheumatic diseases. The primary objective is to evaluate the safety of therapeutic agents. Key secondary objectives are to document the clinical courses and drug treatment patterns of patients and to evaluate clinical outcomes and their determinants, including treatment and other factors. This abstract describes the clinical characteristics of the patients in the CARRA Registry at the time of enrollment.

**Methods**

The CARRA Registry enrolled patients with juvenile idiopathic arthritis (JIA) from CARRA pediatric rheumatology care centers throughout the United States. To facilitate cohort studies within the Registry, there was preferential enrollment of patients who were newly diagnosed or who newly initiated treatment with methotrexate or a biologic agent. Patients with systemic JIA were also preferentially enrolled. Following the initial launch of the Registry, patients with a history of at least 5 joints affected were preferentially enrolled as well. Data at enrollment for all patients enrolled prior to December 31, 2015 were included. Missing values were ignored.

**Results**

Up to December 31, 2015, there were 445 patients enrolled in the CARRA Registry from 41 different CARRA centers. The clinical characteristics of the patients at enrollment are shown. With respect to cohort establishment, 105 patients were enrolled within 6 months of their diagnosis of JIA and 96 patients initiated methotrexate or biologic therapy at the time of enrollment. The numbers of patients currently using methotrexate and specific biologic agents at enrollment are shown.

**Conclusions**

The CARRA Registry successfully enrolled 445 patients with JIA during the first 6 months of operation while the number of activated sites was still accumulating. The current enrollees are enriched for early disease and recent medication initiations, making them ideal subjects for highly informative observational studies. Prospectively collected data from the CARRA Registry will enable rigorous and robust real-world studies of medication safety and disease outcomes.

## P8 Comparative performance of small and large clinical centers in a comprehensive pediatric rheumatology disease registry

### Peter R Blier^1,2^

#### ^1^Baystate Children's Hospital, Springfield, MA, USA; ^2^Tufts University School of Medicine, Boston, MA, USA

##### **Correspondence:** Peter R Blier (peter.blier@bhs.org) – Baystate Children's Hospital, Springfield, MA, USA; Tufts University School of Medicine, Boston, MA, USA

**Background**

Little information is available on the relative ability of researchers at smaller institutions to participate in clinical research. A perception exists that it is less possible to be successful in that setting than at larger centers. In pediatric rheumatology a large fraction of specialists and patients are in small center settings. Advancement of the field would be enhanced by the inclusion of more investigators and more patients.

**Methods**

The CARRA Registry, intended to create a comprehensive registry of all patients with pediatric rheumatic disease, provided support to develop research infrastructure at small as well as large centers. Throughout the 3-year active history of the Registry, monthly updates compiled information about numbers of enrolling centers, new subject enrollments and data collection, follow up visit data collection, and completeness of collected clinical information forms.

We compared the performance of investigators at small and large centers participating in the CARRA Registry. Information at several time points was separated by center size, and totals and per-investigator averages were calculated. Additionally, a compilation of scientific publications based on Registry data was analyzed for authorship, and number of co-authors from small and large centers was compared.

**Results**

9560 total subjects were enrolled in the CARRA Registry at the 59 included participating sites, and data were collected from a total of 41127 patient visits. Small pediatric rheumatology centers comprised 42 % of enrolling sites and 24 % of enrolling investigators. 2416 or 24 % of all enrolled subjects came from small centers. There were no differences in per-investigator performance between providers at small or large centers in any of the data collection parameters measured: number of new enrollments; number of visits collected per patient; or completeness (quality) of the submitted data forms. The results were comparable at all time points evaluated. The pace of collection slowed toward the end of the study period, but the rate was the same between small and large centers. A difference was observed in the number of co-authors from small or large centers on publications arising from the CARRA Registry; investigators from small centers were relatively under-represented (only 14.3 % from small centers vs. 85.7 % from large).

**Conclusions**

Investigators at smaller centers can contribute data of equal quantity and quality to that obtained at institutions with more established research infrastructures. The support CARRA provided in laying the groundwork for research participation at small centers may have contributed to enhancing their inclusion in the registry. However, small-center investigators were under-represented in publications arising from the Registry. The reasons are unclear, but the missed opportunity to achieve academic rewards from research participation may contribute to the perception among investigators at small centers that research is less possible in that setting.

## P9 Clinical characteristics of children with membranous lupus nephritis: The Childhood Arthritis and Rheumatology Research Alliance Legacy Registry

### Alexis Boneparth^1^, Scott E. Wenderfer^2^, L. Nandini Moorthy^1^, Suhas M. Radhakrishna^3^, Anna Carmela P. Sagcal-Gironella^2^, Emily von Scheven^4^

#### ^1^Rutgers-Robert Wood Johnson Medical School, New Brunswick, NJ, USA; ^2^Baylor College of Medicine, Houston, TX, USA; ^3^Rady Children’s Hospital, San Diego, CA, USA; ^4^University of California at San Francisco, San Francisco, CA, USA

##### **Correspondence:** Alexis Boneparth (alexis.boneparth@rutgers.edu) – Rutgers-Robert Wood Johnson Medical School, New Brunswick, NJ, USA

**Background**

Patients with membranous lupus nephritis (MLN) make up 8-30 % of pediatric LN cases. For patients diagnosed initially with pure class V LN, risk of progression to proliferative LN is difficult to ascertain, given variable treatment practices and the limited availability of data from repeat renal biopsies. Although consensus guidelines for management of LN have been formulated, these recommendations are based on evidence from adult studies, and treatment of pediatric LN is largely empirical. The degree of variability in current treatment practices for patients with pediatric class V LN is largely unknown.

**Methods**

Subjects with pediatric systemic lupus erythematosus (SLE) and class V lupus nephritis (LN) from the Childhood Arthritis and Rheumatology Research Alliance (CARRA) Legacy Registry were included. Demographic, disease and medication-related data were collected between 2010 and 2014 from 59 CARRA Legacy Registry sites. Data were utilized to describe demographic, clinical, and treatment characteristics in this cohort.

**Results**

A total of 132 subjects had MLN, either in isolation or in combination with proliferative LN. Seventy-four subjects had pure class V LN. The proportion of subjects with daily corticosteroid treatment was similar among groups (96 %, 91 %, and 96 %, for class III + V, IV + V, and V, respectively, *P* = 0.67). Proportion of subjects exposed to mycophenolate was significantly different among groups, with a trend toward more frequent mycophenolate exposure in the pure class V group (83 %, 74 %, 92 % for class III + V, IV + V, and V, respectively, *P* = 0.045). Proportion of subjects exposed to any disease-modifying antirheumatic drug (DMARD) or biologic was similar among the three groups (83 %, 91 %, 95 % for class III + V, IV + V, and V, respectively, *P* = 0.189). Proportion of subjects with decreased glomerular filtration rate (less than 90 ml/min/1.73 m^2^ ) was significantly different among groups (4 %, 38 %, and 4 %, for class III + V, IV + V, and V, respectively, *P* < 0.0001).

**Conclusions**

To date, this is the largest reported cohort of children with MLN. Practice patterns may vary among centers and may not reflect the published LN consensus treatment guidelines. More research is needed to confirm the trends observed in this analysis of the CARRA Legacy Registry data.

## P10 Rituximab use in pediatric lupus anticoagulant hypoprothrombinemia syndrome - a two center experience

### Kader Cetin Gedik^1^, Salma Siddique^2^, Cassyanne L. Aguiar^1^, Doruk Erkan^2^

#### ^1^Cohen Children’s Medical Center of New York, NY, USA; ^2^Hospital for Special Surgery-Weill Cornell Medical Center, New York, NY, USA

##### **Correspondence:** Kader Cetin Gedik (kcetin@northwell.edu) – Cohen Children’s Medical Center of New York, NY, USA

**Background**

Lupus anticoagulant hypoprothrombinemia syndrome (LA-HPS) is a rare condition that may predispose to both thrombosis and bleeding due to positive lupus anticoagulant (LA) and factor II deficiency. The syndrome is more common in the pediatric population in association with systemic lupus erythematosus (SLE); the majority of patients require immunosuppressive medications as a steroid-sparing agent. The purpose of this case series was to report the use of RTX in the treatment of LA-HPS in pediatric SLE (pSLE).

**Methods**

Medical records of pSLE (≤18 years old) cases that fulfilled American College of Rheumatology (ACR) criteria and were treated with RTX for LA-HPS after glucocorticoid (GC) induction at both institutions were reviewed retrospectively from January 2010 to February 2016. Information obtained in a systematic fashion included demographics, laboratory findings, clinical course, RTX dose/duration, medications, pre/post RTX LA, pre/post RTX factor II levels and outcomes at 1 year follow-up.

**Results**

As of February 2016, we identified 3 cases. All cases presented with bleeding (one with additional thrombosis) and were treated with pulse or high dose methylprednisolone and RTX with complete or clinical resolution of LA-HPS at 1 year follow-up.

**Conclusions**

Our systematic review of the 3 pSLE LA-HPS cases suggests that RTX: a) is an effective steroid sparing agent for LA-HPS; b) does not affect LA positivity; c) improves or normalizes Factor II levels.

## P11 Predictors of complementary and alternative medicine use and response in children with musculoskeletal conditions

### Ezra Cohen^1^, Yvonne Lee^2^, Michelle Dossett^3^, Darshan Mehta^3^, Roger Davis^4^

#### ^1^Boston Children's Hospital, Boston, MA, USA; ^2^Brigham and Women's Hospital, Boston, MA, USA; ^3^Massachusetts General Hospital, Boston, MA, USA; ^4^Beth Israel Deaconess, Boston, MA, USA

##### **Correspondence:** Ezra Cohen (ezra.cohen@childrens.harvard.edu) – Boston Children's Hospital, Boston, MA, USA

**Background**

Complementary and alternative medicines (CAM) are widely used in the general pediatric population, and are disproportionately used in children with musculoskeletal (MSK) conditions. In this study, we identified factors associated with use of CAM therapies in children with MSK conditions. We hypothesized that parental education, parental CAM use and a history of depression, anxiety or stress would be associated with child CAM use.

**Methods**

We analyzed data from the 2012 National Health Interview Survey, including the child CAM use supplement. We defined musculoskeletal conditions as the presence of arthritis, sprain, and back, neck, muscle, bone, or joint pain, or use of CAM therapies for these conditions. We constructed a logistic regression model with use of CAM therapy as the outcome and the following covariates and predictors: age, sex, region, race, school days missed, health status, insurance category, presence of anxiety, depression or stress, highest educational status of one parent, parental CAM use, and ratio to poverty threshold. We also analyzed perceived usefulness of CAM therapies for MSK conditions.

**Results**

The total number of children with MSK conditions was 1,489, representing 4,161,797 U.S. children. 49 % were female with a mean age of 13.2. Controlling for multiple covariates in a logistic regression model, many factors were positively associated with CAM use: presence of depression, anxiety, or stress (OR 1.35, p = 0.028), parental CAM use (OR 2.80, p <0.001), parental education higher than high school (OR 4.59, p <0.001), female status (OR 1.51, p = 0.004), and location in the Western region of the U.S (OR 1.74, p =0.027). Black race was inversely associated with CAM use (OR 0.35, p <0.001). Furthermore, a majority of children (63.5 %) felt that CAM use helped their condition a great deal, and 89.7 % felt that CAM use helped a great deal or some.

**Conclusions**

Certain demographic factors and the presence of depression, anxiety or stress are associated with CAM use in children with MSK conditions. These children appear to have a high rate of response, though sample size was insufficient to compare statistically to other conditions. In the future, understanding if (and if so, which) children with MSK conditions are more responsive to CAM therapies might help to guide management of their symptoms.

## P12 Comparison of pediatric rheumatology and nephrology survey results for the treatment of refractory proliferative lupus nephritis and renal flare in juvenile SLE

### Mileka Gilbert^1^, Beatrice Goilav^2^, Esra Meidan^3^, Joyce Hsu^4^, Alexis Boneparth^5^, Anabelle Chua^6^, Stacy Ardoin^7^, Scott E. Wenderfer^8^, Emily Von Scheven^9^, Natasha M. Ruth^1^

#### ^1^Medical University of South Carolina, Charleston, SC, USA; ^2^Children's Hospital at Montefiore, Bronx, NY, USA; ^3^Boston Children's Hospital, Boston, MA, USA; ^4^Stanford University, Stanford, CA, USA; ^5^Rutgers University, Rutgers, NJ, USA; ^6^Duke University, Durham, NC, USA; ^7^Nationwide Children's Hospital, Columbus, OH, USA; ^8^Baylor College of Medicine, Houston, TX, USA; ^9^UCSF, San Francisco, CA, USA

##### **Correspondence:** Mileka Gilbert (gilbertm@musc.edu) – Medical University of South Carolina, Charleston, SC, USA

**Background**

Consensus treatment plans (CTPs) for induction treatment of newly diagnosed proliferative lupus nephritis (LN) in juvenile SLE have been developed to alleviate some of the decision-making burden and heterogeneity of this disease. However, patients who do not fully respond to any of the regimens or develop disease flare after remission require escalation of treatment. In this study, we took the first step in developing CTPs for refractory proliferative LN and flare by determining current practices of pediatric nephrologists and rheumatologists in US and Canada.

**Methods**

Members of CARRA and the American Society for Pediatric Nephrology (ASPN) were surveyed in November 2015 to assess treatment choices (other than modifying steroid dosing) and level of agreement between rheumatologists and nephrologists for proliferative LN patients. 2 cases were presented: 1) patients refractory to induction treatment with corticosteroid and cyclophosphamide (CYC) and 2) patients who flare after initial response to induction.

**Results**

75 nephrologists and 37 rheumatologists completed the refractory LN case survey questions. There were 3 scenarios at 7 months into treatment including elevated creatinine with persistent hematuria and nephrotic syndrome; nephrotic syndrome with mild hematuria and improved creatinine; and persistent hematuria with improved creatinine and proteinuria. In the scenario of elevated creatinine at follow up, 44 % of rheumatologists and 42 % of nephrologists agreed with the top choice of mycophenolate mofetil (MMF) + rituximab (RTX). CYC + RTX (27 % vs. 16 %) and MMF alone (20 % vs. 20 %) were the next most common choices for therapy in this situation. In the scenario of persistent proteinuria, both rheumatologists and nephrologists chose MMF alone (37 % vs. 41 %) and MMF + RTX (34 % vs. 15 %). In the scenario of persistent hematuria, both rheumatologists and nephrologists chose MMF alone (38 % vs. 59 %) and MMF + RTX (35 % vs. 11 %)[Ev1] 63 nephrologists and 33 rheumatologists completed the survey questions for the LN case for flare after achieving remission on induction therapy. For LN flare after CYC induction, rheumatologists chose CYC + RTX (36 %) or CYC alone (21 %), whereas nephrologists chose CYC alone (22 %) or increased the MMF (22 %). For LN flare after MMF induction, rheumatologists chose CYC (27 %) or MMF + RTX (24 %), and nephrologists chose increased MMF (38 %) or CYC (23 %). For LN flare with renal insufficiency and biopsy-confirmed rapidly progressive GN with significant activity and chronicity, rheumatologists and nephrologists agreed that CYC with or without RTX (78 % vs. 65 %) was the best therapeutic choice, although more rheumatologists would use combination CYC + RTX (57 % vs. 22 %).

**Conclusions**

Pediatric rheumatologists and nephrologists agree on treatment of severe cases of proliferative LN refractory to induction therapy or LN flare after remission. However, there were differences in choice of therapies in the milder cases, specifically in the use of rituximab. Further investigation will be necessary to delineate the reasons behind this finding. This study highlights the importance of collaborative effort in developing CTPs for pediatric LN.

## P13 Transitioning lupus patients from pediatric to adult rheumatology

### Joyce Hui-Yuen^1^, Kader Cetin Gedik^1^, Liza Bermudez^2^, Ashlea Cook^2^, Lisa Imundo^3^, Amy Starr^2^, Andrew Eichenfield^2^, Anca Askanase^3^

#### ^1^Division of Pediatric Rheumatology, Cohen Children’s Medical Center, New Hyde Park, NY, USA; ^2^Division of Pediatric Rheumatology, Morgan Stanley Children’s Hospital of New York-Presbyterian, Columbia University Medical Center, New York, NY, USA; ^3^Division of Adult Rheumatology, Columbia University Medical Center, New York, NY, USA

##### **Correspondence:** Joyce Hui-Yuen (jhuiyuen@nshs.edu) – Division of Pediatric Rheumatology, Cohen Children’s Medical Center, New Hyde Park, NY, USA

**Background**

Pediatric rheumatologists have successfully improved the life expectancy and quality of life of children with systemic lupus (cSLE). cSLE has higher disease severity and morbidity than adult SLE, resulting in the need to address transition to Adult Rheumatology. The consequences of poor transition are loss of continuity in care and medication regimens resulting in worse disease activity and damage. As data are scarce on successful transition of cSLE patients to adult care, we conducted a pilot study to better understand how to facilitate transition of cSLE patients to adult care.

**Methods**

Patients with cSLE ≥14 years old who fulfilled ACR SLE criteria were evaluated using a 29-item Transition Readiness Questionnaire (SLE-TRQ) adapted from cystic fibrosis and sickle cell transitioning questionnaires. It included skills (medical and life skills), knowledge (medical and independent living), and psychological factors (feelings, stress about transition). A protocol based on the American Association of Pediatrics guidelines for successful transition was implemented for patients ≥18 years old. All patients met with the adult rheumatologist in the pediatric clinic and were accompanied to their first visit to the adult clinic by the Pediatric Rheumatology nurse. A successful transition was defined as ≥ 3 visits with the adult rheumatologist. All successfully transitioned patients were asked to complete a Satisfaction Questionnaire.

**Results**

40 patients with a mean age of 18.4 years (range 14-26) completed the SLE-TRQ. The mean disease duration was 5.25 years; 75 % were female; 45 % Hispanic, 40 % African American, 7 % Caucasian. There were 93 % who had major organ involvement, 19 (48 %) renal disease, 8 (20 %) neuropsychiatric lupus, 3 (10 %) abnormal pulmonary function tests and 2 (5 %) anti-phospholipid syndrome. Eleven patients were on prednisone, median dose 20 mg/day (2.5-60 mg/day) at the time of enrollment. Over 50 % were noncompliant at least on one occasion with medications and appointments. For the SLE-TRQ, 27 patients self-reported good medical and independent living skills, 14 good knowledge, and 16 uneasiness/unreadiness for transition. The mean scores were: skills- 1.39, knowledge- 2.4 and feelings about transition- 2.3, where scores of 4-5 identify patients that are not ready, 2-3 patients in preparation and 0-1 ready for transition. Eight patients remain under pediatric care, scheduled to transition at a later date. Of the 20 patients scheduled to transition, 12 successfully transferred. The mean SLE disease activity index at the last pediatric visit was 5.6 and at the third adult visit 5.25. Three patients were on prednisone (mean dose 13.8 mg/day). Nine patients completed the Satisfaction Questionnaire, reporting satisfaction with the transition process.

**Conclusions**

This is the first study evaluating self-reported pre-transition readiness in cSLE. Patients had good medical knowledge and independent living skills but some were not ready to transition. We were able to successfully transition 60 % of the patients suggesting adolescents with cSLE and major organ involvement need additional support in the transition to adult rheumatology.

## P14 The systemic juvenile idiopathic arthritis cohort of the Childhood Arthritis & Rheumatology Research Alliance Registry

### Ginger Janow^1^, Laura E. Schanberg^2,3^, Soko Setoguchi^3^, Victor Hasselblad^3^, Elizabeth D. Mellins^4^, Rayfel Schneider^5^, Yukiko Kimura^1^, The CARRA Legacy Registry Investigators^3^

#### ^1^Pediatrics, Joseph M Sanzari Children's Hospital, Hackensack, NJ, USA; ^2^Pediatrics, Duke University, Durham, NC, USA; ^3^Duke Clinical Research Institute, Durham, NC, USA; ^4^Pediatrics, Stanford University, Stanford, USA; ^5^Pediatrics, Hospital for Sick Children and University of Toronto, Toronto, Canada

##### **Correspondence:** Ginger Janow (gjanow@hackensackumc.org) – Pediatrics, Joseph M Sanzari Children's Hospital, Hackensack, NJ, USA

Elizabeth D. Mellins, Rayfel Schneider and Yukiko Kimura contributed equally to this work.

**Objectives**

We aimed to identify (1) demographic/clinical characteristics; (2) medication usage trends; (3) variables associated with worse disease activity; and (4) characteristics of patients with persistent chronic arthritis, in the Childhood Arthritis and Rheumatology Research Alliance (CARRA) legacy Registry systemic JIA cohort.

**Methods**

Demographics, disease activity measures and medications at enrollment of systemic JIA patients in the CARRA Registry were analyzed using descriptive statistics. Multivariate analyses were conducted to identify associations with increased disease activity. Medication usage frequencies were calculated by year.

**Results**

528 systemic JIA patients were enrolled (2010-2013). 435 patients had a complete dataset; of these, 372 met ILAR criteria and were included in the analysis. At enrollment, median disease duration and joint count were 3.7 years and 0 respectively; 16.4 % had rash and 6.7 % had fever. 26 % were taking IL-1 inhibitors and 29 % glucocorticoids. DMARD and TNF inhibitors use decreased, while IL-6 inhibitor use increased between 2010 and 2013. African-American (AA) patients had worse joint counts (p = 0.003), functional status (p = 0.01) and physician global assessment (p = 0.008). Of 255 subjects with >2 years disease duration, 56 % had no arthritis or systemic symptoms, while 32 % had persistent arthritis only.

**Conclusions**

Most patients in the largest systemic JIA cohort reported to date had low disease activity. Practice patterns for choice of biologic agents appeared to change over the study period. Nearly 1/3 had persistent arthritis without systemic symptoms >2 years after onset. AA race was associated with worse disease activity. Strategies to improve outcomes in subgroups with poor prognosis are needed.

## P15 Results of the pilot study of the Childhood Arthritis and Rheumatology Research Alliance (CARRA) consensus treatment plans for new-onset systemic juvenile idiopathic arthritis

### Yukiko Kimura^1^, Sriharsha Grevich^2^, Timothy Beukelman^3^, Esi Morgan^4^, T Brent Graham^5^, Maria Ibarra^6^, Yonit Sterba Ruas^7^, Marisa Klein-Gitelman^8^, Karen Onel^9^, Sampath Prahalad^10^, Marilynn Punaro^11^, Sarah Ringold^2^, Dana Toib^12^, Heather Van Mater^13^, Jennifer E. Weiss^1^, Pamela F. Weiss^14^, Kelly Mieszkalski^15^, Laura E. Schanberg^13^

#### ^1^Hackensack University Medical Center, Hackensack, NJ, USA; ^2^Seattle Children's Hospital, Seattle, WA, USA; ^3^University of Alabama, Tuscaloosa, AL, USA; ^4^Cincinnati Children's Hospital Medical Center, Cincinnati, OH, USA; ^5^Vanderbilt University, Vanderbilt, TN, USA; ^6^Children's Mercy Hospital, Kansas City, MO, USA; ^7^Children's Hospital of Montefiore, Bronx, NY, USA; ^8^Lurie Children's Hospital of Chicago, Chicago, IL, USA; ^9^Comer Children's Hospital of Chicago, Chicago, IL, USA; ^10^Emory Children's Hospital, Atlanta, GA, USA; ^11^Texas Scottish Rite Hospital, Dallas, TX, USA; ^12^St. Christopher's Hospital for Children, Philadelphia, PA,USA; ^13^Duke University, Durham, NC, USA; ^14^Children's Hospital of Philadelphia, Philadelphia, PA, USA; ^15^ CARRA, Durham, NC, USA

##### **Correspondence:** Yukiko Kimura (ykimura@hackensackumc.org) – Hackensack University Medical Center, Hackensack, NJ, USA

**Background:**

Systemic Juvenile Idiopathic Arthritis (systemic JIA) is commonly treated with glucocorticoids (GC) alone, or methotrexate (MTX) or biologic medications (most commonly IL1 or IL6 inhibitors), alone or in combination with GC. The most effective treatment for new onset systemic JIA is not known. The Childhood Arthritis and Rheumatology Research Alliance (CARRA) developed standardized consensus treatment plans (CTPs) of these therapies for untreated systemic JIA. These CTPs will be used to better understand the comparative effectiveness of these treatments. A pilot study was conducted to assess the feasibility of using the CTPs for this purpose using an observational registry.

**Methods:**

New onset untreated systemic JIA patients (pts) were enrolled in the CARRA Registry and treated using the CTP selected by the treating physician and patient/family (GC alone; MTX ± GC; IL1 inhibitor (IL1i) ± GC; IL6 inhibitor (IL6i) ± GC. An aggressive taper was suggested if GC was started, with the goal of reducing the dose to <50 % of the starting dose by 3 mos. Standard of care intervals were used as data collection time points. The primary outcome was clinically inactive disease (CID) off all GC at the 9 month visit. The CTPs were compared using chi square, Fisher’s exact, and Wilcoxon rank sum tests.

**Results:**

30 patients were enrolled at 13 sites. 8 were started on a non-biologic (2 GC, 6 MTX) and 22 on a biologic (12 IL1i and 10 IL6i). One patient was lost to follow up. CTP choice appeared to segregate primarily by site. No major differences in demographic or disease features were found between each CTP group. The primary outcome was achieved by 41 % of patients overall (11 of 27) on whom we have 9 month visit data. 0 of 7 patients started on a non-biologic CTP (GC or MTX) achieved the outcome, while 11 of 20 (55 %) of those started on a biologic CTP (IL1i and IL6i) did (p = 0.022). There were 3 serious adverse events: 2 infections requiring hospitalization while receiving IL1i (1 patient was on GC and had cellulitis; the other was hospitalized for Grade 2 varicella and treated with IV acyclovir as a precaution) and 1 macrophage activation syndrome (MAS) requiring hospitalization (on IL6i). There was an additional Grade 2 MAS episode in an IL1i treated patient. All patients recovered without sequelae.

**Conclusions:**

The CARRA systemic JIA CTP pilot study was completed successfully. The patients were reasonably balanced at baseline between CTP groups, and the primary determinants of CTP choice appeared to be site and physician preference rather than patient characteristics. Those initially treated with biologics had a greater likelihood of achieving clinical inactive disease off GC at 9 months. Having demonstrated feasibility of the CTPs, additional patients will be enrolled as part of a larger comparative effectiveness study to determine the most appropriate treatment strategy for new onset systemic JIA.

**Reference**

[1] Y Kimura, T Beukelman, E Morgan-DeWitt, KL Mieszkalski, TB Graham, MF Ibarra, N Ilowite, MS Klein-Gitelman, K Onel, S Prahalad, MG Punaro, S Ringold, D Toib, H Van Mater, PF Weiss, L Schanberg and the CARRA Registry Investigators. Results from the Childhood Arthritis and Rheumatology Research Alliance Systemic JIA Consensus Treatment Plans Pilot Study. Arthritis Rheumatol 2015;67;Suppl 10: abstract 959

## P16 A systemic review of pain relief modalities in juvenile idiopathic arthritis: First step in developing a novel decision support intervention

### Timothy S. H. Kwok^1^, Jacinthe Bisaillon^2^, Christine Smith^3^, Lucie Brosseau^4^, Jennifer Stinson^5^, Adam M. Huber^6^, Ciaran M. Duffy^7^, Karine Toupin April^8^

#### ^1^Undergraduate Medical Education, Faculty of Medicine, University of Ottawa, Ottawa, Ontario, Canada; ^2^School of Nursing Sciences, Faculty of Health Sciences, University of Ottawa, Ottawa, Ontario, Canada; ^3^School of Epidemiology, Public Health and Preventive Medicine, Faculty of Medicine, University of Ottawa, Ottawa, Ontario, Canada; ^4^School of Rehabilitation Sciences, Faculty of Health Sciences, University of Ottawa, Ottawa, Ontario, Canada; ^5^Hospital for Sick Children, Lawrence S. Bloomberg Faculty of Nursing University of Toronto, Toronto, Ontario, Canada; ^6^IWK Health Centre, Department of Pediatrics, Dalhousie University, Halifax, Nova Scotia, Canada; ^7^Department of Pediatrics, Faculty of Medicine, University of Ottawa, Children's Hospital of Eastern Ontario Research Institute, Ottawa, Ontario, Canada; ^8^Children's Hospital of Eastern Ontario Research Institute, Department of Pediatrics, Faculty of Medicine, School of Rehabilitation Sciences, Faculty of Health Sciences, University of Ottawa, Ottawa, Ontario, Canada

##### **Correspondence:** Timothy S.H. Kwok (tkwok016@uottawa.ca) – Undergraduate Medical Education, Faculty of Medicine, University of Ottawa, Ottawa, Ontario, Canada

**Background**

In juvenile idiopathic arthritis (JIA), although mainstay pharmacological therapy is useful in reducing disease activity, pain often persists in a subset of patients. As such, effective pain management utilizes a multidisciplinary approach, involving pharmacological, physical, nutritional and psychological modalities. Due to the complexity in pain relief modalities, there is a need for an intervention to help youth with JIA and their parents make informed decisions that are consistent with their values and preferences regarding these treatments. The first step in creating a robust evidence-based patient decision aid is to systematically review the evidence for these modalities in JIA.

**Methods**

Major databases (Medline, Embase, PsycInfo, *the Cochrane Library* and AMED) were searched for randomized controlled trials (RCTs) of interventions in JIA compared to any control group that used pain as an outcome measure from inception of the databases to the end of May 2015. For each study, the mean change in pain intensity and standard deviation in each study arm was extracted and the standardized mean difference (SMD) was calculated using Review Manager.

**Results**

361 citations were found after removal of duplicates. A total of 18 RCTs were included. RCTs were found for massage, splints and orthoses, therapeutic exercises, psychosocial modalities, non-steroidal anti-inflammatory drugs (NSAIDs), disease-modifying anti-rheumatic drugs (DMARDs) and biologics. Effective non-pharmacological pain relief modalities included massage compared with relaxation therapy (SMD = 2.88 (1.55,4.2)), custom-made orthoses compared with off-the-shelf shoe inserts (SMD = 1.66 (0.76,2.56)) and with supportive shoes (SMD = 1.55 (0.68,2.41)), pilates compared with a conventional exercise program (SMD = 1.5 (0.86,2.13)), fitted foot orthoses (FOs) with customized chair-side corrections compared with FOs without any corrections (SMDs = 0.86 (0.33,1.39)) and an internet-based self-management program compared with telephone support (SMD = 0.81(0.21,1.41)). Effective pharmacological pain relief modalities included DMARDS (e.g., methotrexate, D-penicillamine, hydroxychloroquine) compared with placebo (SMDs = 0.44 (0.06, 0.82) to 0.76 (0.36, 1.16)) and biologics (e.g., tocilizumab, abatacept) compared with placebo (SMDs = 0.92 (0.54, 1.29) to 1.15 (0.5, 1.81)).

**Conclusions**

This systematic review shows that a wide variety of modalities are effective in reducing pain in JIA. However, high heterogeneity in patient characteristics, treatment regimen, as well as control interventions in trials underlines the importance of discussing the relevance of the evidence before including it in a decision aid. The next step aims to conduct a consensus meeting with patients, clinicians and researchers to determine the content and format of the decision aid, including its evidence-based information.

## P17 Barriers and facilitators to care retention for pediatric systemic lupus erythematous patients in South Africa: A qualitative study

### Laura B Lewandowski^1,2,3,4^, Christiaan Scott^3^

#### ^1^Pediatric Rheumatology, Duke University Medical Center, Durham, NC, USA; ^2^Duke Global Health Institute, Duke University, Durham, NC, USA; ^3^Paediatric Rheumatology, Red Cross War Memorial Children’s Hospital and University of Cape Town, Cape Town, South Africa; ^4^National Institute of Arthritis, Musculoskeletal, and Skin Diseases, NIH, Bethesda, MD, USA

##### **Correspondence:** Laura B Lewandowski (laura.lewandowski@nih.gov) – Pediatric Rheumatology, Duke University Medical Center, Durham, NC, USA; Duke Global Health Institute, Duke University, Durham, NC, USA; Paediatric Rheumatology, Red Cross War Memorial Children’s Hospital and University of Cape Town, Cape Town, South Africa; National Institute of Arthritis, Musculoskeletal, and Skin Diseases, NIH, Bethesda, MD, USA

**Background**

Systemic Lupus Erythematosus (SLE) is a multisystem chronic autoimmune illness. In South Africa (SA), pediatric SLE (pSLE) patients have worse outcomes compared to peers in North America and require specialized medical care with long term follow up. The aim of this study was to describe caregivers’ experiences engaging in chronic medical care for SLE, and to identify barriers and facilitators to patient retention in care. Findings from this study can inform interventions to improve chronic care of pediatric SLE patients in SA.

**Methods**

In-depth, semi-structured interviews were conducted with 22 caregivers of pediatric SLE patients recruited from three centers in Cape Town, SA in 2014. Interviews were audio-recorded, transcribed, and analyzed for themes related to barriers to diagnosis.

**Results**

Four themes were identified and classified under caregiver or health system barriers to chronic care engagement and retention, and three themes were identified as facilitators. Barriers identified were a knowledge gap regarding SLE, financial barriers to care, and the social stigma of SLE, and the complexity of the SA medical system. Chronic health care facilitators identified were patient/parent education, a robust support system for caregiver, and financial support for the patient and caregiver.

**Conclusions**

Caregivers in this study reported challenges in returning for long term care of SLE in their children. Improving family education at diagnosis and visits may increase patient-physician trust, empower patients and caregivers, and motivate them to return for care. Supplementing travel cost for families may mitigate the largest financial and time burden and improve patient retention.

## P18 Evaluating the feasibility of conducting comparative effectiveness studies in juvenile Localized Scleroderma (jLS)

### Suzanne C. Li^1^, Kathryn S. Torok^2^, C. Egla Rabinovich^3^, Sandy D. Hong^4^, Mara L Becker^5^, Fatma Dedeoglu^6^, Maria F. Ibarra^5^, Polly J Ferguson^4^, Rob C. Fuhbrigge^6^, Katie G. Stewart^7^, Elena Pope^8^, Ronald M. Laxer^8^, Thomas G. Mason^9^, Gloria C. Higgins^10^, Xiaohu Li^11^, Marilynn G. Punaro^7^, George Tomlinson^11^, Eleanor Pullenayegum^11^, John Matelski^11^, Laura Schanberg^3^, Brian M. Feldman^8^

#### ^1^Hackensack University Medical Center, Hackensack, NJ, USA; ^2^Children’s Hospital of Pittsburgh, UPMC, Pittsburgh, PA, USA; ^3^Duke University, Durham, NC, USA; ^4^University of Iowa, Iowa City, IA, USA; ^5^Children’s Mercy Hospital, Kansas City, MO, USA; ^6^Boston Children's Hospital, Boston, MA, USA; ^7^Texas Scottish Rite Hospital, Dallas, TX, USA; ^8^Hospital for Sick Kids, Toronto, Canada; ^9^Mayo Clinic, Rochester, MN, USA; ^10^Nationwide Children's Hospital, Columbus, OH, USA; ^11^Stevens Institute of Technology, Hoboken, NJ, USA

##### **Correspondence:** Suzanne C. Li (sli@hackensackumc.org) – Hackensack University Medical Center, Hackensack, NJ, USA

**Background**

Aim: To evaluate the feasibility of conducting comparative effectiveness studies in jLS and identify potential confounders for evaluating treatment response. JLS is a rare chronic disease that often causes major morbidity for the growing child including hemiatrophy, arthropathy, and seizures.

**Methods**

We conducted a prospective observational cohort study of juvenile localized scleroderma subjects who were beginning treatment with one of 3 standardized regimens (consensus treatment plans [CTPs]). The CTPs were: methotrexate alone, methotrexate with intravenous pulse methylprednisolonge, and methotrexate with oral corticosteroids. Choice of CTP was up to the subject’s physician. Subjects were evaluated with standardized activity and damage assessment forms at 6 visits over 1 year. The majority of the data was entered into the web-based legacy CARRA registry; some paper study forms were collected to aid with evaluating clinical assessment tools and additional patient and parent health related quality of life measures. The primary outcome was to assess the feasibility of enrolling patients. Secondary aims were to explore the performance of developed clinical tools for evaluating clinical state and treatment response, and assess the value of other HRQOL questions. Data was analyzed by descriptive statistics, correlation analyses, factor analysis, linear regression, and Bayesian methods.

**Results**

Physicians at 10 CARRA sites enrolled between 1 and 9 subjects (median 5.5) into all 3 CTPs. The target enrollment of 50 subjects was reached, with enrollment finishing 26 months after study initiation. The actual enrollment period was 16 months, with initiation of enrollment at each site delayed for 10-17 months by time required for IRB and contract approval. The average rate of subject accrual was 3.1 subjects/month during the active enrollment period. There were no significant differences between subjects in the different CTPs for age, gender, race, or ethnicity; most were white, non-hispanic girls with a median age of 13 years, and median disease duration of 13 months. Most were new to treatment with systemic immunosuppressants. At baseline, over 70 % of subjects had extracutaneous involvement, with 32 % having joint involvement and 46 % having a growth difference. Significant differences were found between subjects in the different CTPs for disease duration, prior treatment with systemic immunosuppressants, LS subtype, and some types of extracutaneous involvement.

**Conclusions**

We achieved our primary outcome of showing the feasibility of conducting comparative effectiveness studies in jLS, a rare disease. We reached our target enrollment and enrolled subjects into all 3 CTPs. We identified a much higher rate of extracutaneous and severe morbidity than has previously been reported, possibly related to detailed prospective data collection. Identifying potential confounders will help determine appropriate sample size for conducting full-scale jLS comparative effectiveness studies.

**Acknowledgements**

The study received funding from Arthritis Foundation, NIAMS, DCRI, and Friends of CARRA.

## P19 Tonsillar histology in patients with periodic fever, aphthous stomatitis, pharyngitis, adenitis (PFAPA) syndrome

### Kalpana Manthiram^1^, Hernan Correa^2^, Kathryn Edwards^1^

#### ^1^Division of Pediatric Infectious Diseases, Vanderbilt University School of Medicine, Nashville, TN, USA; ^2^Department of Pathology, Immunology, and Microbiology, Vanderbilt University School of Medicine, Nashville, TN, USA

##### **Correspondence:** Kalpana Manthiram (kalpana.manthiram@nih.gov) – Division of Pediatric Infectious Diseases, Vanderbilt University School of Medicine, Nashville, TN, USA

**Background**

Periodic fever, aphthous stomatitis, pharyngitis, and cervical adenitis (PFAPA) syndrome is the most common periodic fever syndrome of childhood, and tonsillectomy is reported to be curative in many patients. The objective of our study was to compare the histologic features of palatine tonsils surgically removed from children with PFAPA and those with obstructive sleep apnea (OSA).

**Methods**

Age-matched patients with PFAPA and OSA who had undergone tonsillectomy at Vanderbilt University Medical Center from 2000-2015 were recruited. Participants’ archival paraffin-embedded, formalin-fixed palatine tonsil tissue blocks were obtained. The following histologic features were measured after hematoxylin and eosin (H&E) staining: average germinal center area, mantle width, interfollicular distance, crypt width, and surface squamous epithelium width. Representative areas of germinal centers, crypts, and surface squamous epithelium were assembled on tissue microarrays, and immunohistochemical stains for lymphoid and myeloid cells were performed. Staining was assessed with digital image analysis. Features of matched PFAPA and OSA tonsils were compared using the Wilcoxon-signed ranks test, and associations of histologic features and time from the last episode were compared with Spearman’s test.

**Results**

Sixteen cases with PFAPA and 16 controls with OSA were recruited. Histologic measurements from PFAPA and OSA tonsils are compared. Tonsils from patients with PFAPA had significantly smaller germinal centers, less variation in germinal center area, and wider surface squamous epithelia than those of patients with OSA. Percentages of B and T lymphocytes, macrophages, and dendritic cells were not significantly different in the germinal centers, crypts, and squamous epithelium of PFAPA and OSA tonsils. Among PFAPA patients, longer time from the last fever episode was associated with larger germinal center area (Spearman’s rho = 0.61, p = 0.02); however, this association did not remain significant with multivariable analysis.

**Conclusions**

Germinal centers in tonsils from patients with PFAPA did not exhibit hyperplasia, but the tonsillar surface squamous epithelium of PFAPA patients was wider compared with children with OSA. In tonsils from PFAPA patients, the germinal centers appeared to enlarge with time after the last episode, suggesting that tonsillar architecture may evolve with time during fever cycles. We plan to further study lymphocyte activation, proliferation, and migration in the tonsils of PFAPA patients to understand the mechanism of these histologic differences.

## P20 Clinical course of juvenile dermatomyositis presenting as skin predominant disease

### Edward J. Oberle^1^, Michelle Bayer^2^, Dominic O. Co^2^, Hatice Ezgi Baris^3^, Yvonne Chiu^2^, Adam Huber^4^ , Susan Kim^3,5^

#### ^1^Nationwide Children's Hospital, The Ohio State University, Columbus, OH, USA; ^2^Children's Hospital of Wisconsin, Medical College of Wisconsin, Milwaukee, WI, USA; ^3^Boston Children's Hospital, Boston, MA, USA; ^4^IWK Health Centre, Dalhousie University, Halifax, NS, Canada; ^5^Harvard Medical School, Boston, MA, USA

##### **Correspondence:** Edward J. Oberle (edward.oberle@nationwidechildrens.org) – Nationwide Children's Hospital, The Ohio State University, Columbus, OH, USA

**Background**

Juvenile dermatomyositis (JDM) is a chronic inflammatory disorder of the skin and striated muscle. A subset of patients can present with rash only, labeled as *skin predominant JDM* (spJDM) for this study. The natural course of patients with spJDM and optimal treatment is unknown. The purpose of this study is to describe the clinical course of spJDM patients and assess for early indicators that may predict progression to classic JDM.

**Methods**

A chart review to identify patients presenting with spJDM was performed on all patients with the diagnosis code for dermatomyositis seen at 3 CARRA sites (Children’s Hospital of Wisconsin, Boston Children’s Hospital, IWK Health Centre). Data collected included patient demographics, presenting symptoms and exam findings, initial treatment, muscle enzymes, muscle biopsy, electromyography, and magnetic resonance imaging (MRI). Patients were categorized as either amyopathic (no weakness on exam and no diagnostic studies consistent with myositis) or hypomyopathic (no weakness but diagnostic studies showed subclinical myositis). Follow up visits were reviewed for development of weakness.

**Results**

Twenty-four patients presented with spJDM: 8 (33 %) were amyopathic on initial evaluation, while 16 (67 %) were hypomyopathic. None of the amyopathic patients later developed weakness (follow up 3-144 months, median 31 months). Six (38 %) hypomyopathic patients later evolved into classic JDM with weakness (follow up 10-85 months, median 45 months). Time to development of weakness ranged from 3 to 24 months after onset of rash. Patients who developed weakness had varying degrees of subclinical myositis as evidenced by lab or MRI. MRI was abnormal in 7 of 20 patients (35 %) at baseline. Only 2 of these 7 patients (29 %) later developed weakness. Patients with an abnormal MRI were more likely to receive systemic corticosteroids and/or methotrexate as part of initial therapy (43 % vs 8 %). The combination of hydroxychloroquine (HCQ) with a topical calcineurin inhibitor was the most common first line agent (21 %). However, whether alone or in combination with a topical agent, HCQ was initiated in 46 % of patients. Nine percent of patients treated with HCQ with or without topical agents developed weakness, while 50 % treated with topical agents alone and 40 % treated with systemic steroids developed weakness. Methotrexate was always started in conjunction with systemic steroids (4/24). Two patients with no initial treatment were later treated with HCQ or topical steroid for persistent skin disease and never had weakness.

**Conclusions**

Our work suggests that topical therapies do not prevent the development of weakness, but systemic treatment with HCQ may diminish the development of weakness in spJDM (50 % vs 9 %). No amyopathic patient progressed to develop weakness, while 38 % of hypomyopathic patients developed weakness. This suggests the importance of comprehensive baseline testing using clinical, lab and MRI assessments, and that hypomyopathic patients should be followed more closely to assess for disease progression over time. Larger trials are needed to identify long term outcomes, optimal treatment, and whether treatment of spJDM prevents weakness.

## P21 A Survey of musculoskeletal ultrasound practices of pediatric rheumatologists in North America

### Edward J Oberle^1,2^, Timothy Beukelman^3^

#### ^1^Nationwide Children's Hospital, Columbus, OH, USA; ^2^The Ohio State University, Columbus, OH, USA; ^3^University of Alabama at Birmingham, Birmingham, AL, USA

##### **Correspondence:** Edward J Oberle (edward.oberle@nationwidechildrens.org) – Nationwide Children's Hospital, Columbus, OH, USA; The Ohio State University, Columbus, OH, USA

**Background**

Musculoskeletal ultrasound (MSUS) is a well-established tool in the diagnosis and management of inflammatory arthritis. The use among pediatric rheumatologists, however, continues to be limited. The purpose of this study was to assess the patterns of MSUS use among North American pediatric rheumatologists and identify barriers impeding the wider acceptance of MSUS.

**Methods**

All Juvenile Idiopathic Arthritis (JIA) committee members of the Childhood Arthritis and Rheumatology Research Alliance (CARRA) from the United States and Canada were invited to complete an anonymous web-based survey designed to evaluate MSUS use. Questions focused on patterns of MSUS training, certification and utilization, and ease of establishing an MSUS-based clinic. Attitudes toward MSUS were solicited from practitioners not performing ultrasound.

**Results**

The survey response rate was 58 of 204 (28 %). Of those pediatric rheumatologists who responded, 25 (43 %) routinely perform MSUS, 5 (9 %) have received training in MSUS but do not actively use it, 16 (28 %) are interested in MSUS, while 12 (20 %) have no interest. All but one (98 %) found MSUS to be a valuable tool for patient care. Active sonographers (N = 25) tend to be within 10 years out of fellowship (60 %) and primarily clinicians based at academic centers (84 %). Duration of clinical practice varied from less than 1 year (28 %), 2-5 years (68 %), and more than 6 years (4 %). Most attended a formal training course (88 %) with American College of Rheumatology (ACR) courses being most common. 10 (40 %) completed extensive training through Ultrasound School of North American Rheumatologists (USSONAR). Exposure in fellowship was limited (28 %). Formal certification through ACR or the Canadian Rheumatology Ultrasound Society (CRUS) was rare (8 % and 4 % respectively). Most rheumatologist sonographers performed US at least weekly (83 %) by incorporating US into routine appointments rather than dedicated US clinics (83 vs 30 %). Primary indications for MSUS included diagnosis synovitis (87 %) and joint injection guidance (83 %). 65 % reported no difficulties starting US in their practice. Main barriers included allocating clinic time for US (13 %) and obtaining a machine (9 %). Institutional or radiology department interference was rare (13 %); most report a supportive or indifferent relationship with radiology (78 %). Only 53 % of sonographers bill for MSUS with 23 % of them reporting difficulty with receiving payment from insurers regardless of certification status. Of non- sonographers, those with interest in MSUS training (N = 16) tend to be in the earlier phase of their career and dependent on radiology to obtain US images rather than a rheumatologist partner who performs MSUS. The major limitations to pursuing MSUS training are the lack of time or financial support for training (75 %) and expense of equipment (62 %).

**Conclusions**

MSUS use and interest among pediatric rheumatologists is likely increasing. Barriers are not as widespread as once perceived. Survey respondents were likely biased towards those who practice MSUS or are supportive of the practice. Nevertheless, more pediatric-specific MSUS training opportunities are needed.

## P22 Assessment, classification and treatment of calcinosis as a complication of juvenile dermatomyositis: A survey of pediatric rheumatologists by the Childhood Arthritis and Rheumatology Research Alliance

### Amir B. Orandi^1^, Kevin W. Baszis^1^, Vikas Dharnidharka^1^, Mark F. Hoeltzel^2^, for the CARRA JDM Committee

#### ^1^St. Louis Children’s Hospital, Washington University School of Medicine, St. Louis, MO, USA; ^2^Mott Children’s Hospital, University of Michigan Medical School, Ann Arbor, MI, USA

##### **Correspondence:** Amir B. Orandi (orandi_a@kids.wustl.edu) – St. Louis Children’s Hospital, Washington University School of Medicine, St. Louis, MO, USA

**Background**

Juvenile dermatomyositis (JDM) is the most common inflammatory myopathy of childhood, with classic manifestations of symmetric proximal muscle weakness and characteristic rash. Despite progress in treatment, calcinosis remains a complication with steady incidence despite improved outcomes in JDM. To date, there is no standardized approach to the management of calcinosis and its various phenotypes. To support the development of a future consensus treatment plan, we sought to characterize physician perspectives of calcinosis as it occurs in JDM. We hypothesized that based on physician experience and preference; there is variability in diagnostic approach, classification, and treatment strategies directly targeting calcinosis (independent of JDM therapy).

**Methods**

After IRB exemption, an electronic survey was created using REDCap, with 23 questions organized by section. Invitations to complete the survey voluntarily and anonymously were sent to CARRA physician members and the Pediatric Rheumatology Bulletin Board, an electronic list-serv. Analysis was by descriptive statistics.

**Results**

Of 139 survey responses, 135 met inclusion criteria by providing clinical care to JDM patients less than 21 years of age. Only 106 fully completed the survey, but 121 completed at least one section in addition to demographics and were included in analysis. Of these, 91 (75 %) were CARRA members, a 30 % response rate. Pediatric rheumatologists comprised 95 % of respondents (U.S.-based totaled 75 % with Europe at 14 %). 29 % of respondents are currently in or within 5 years of fellowship, and 26 % have more than 20 years of experience. The majority (61 %) has treated 10 or less JDM patients with calcinosis and of these, 46 % have less than 15 years of experience. Only 17 % of respondents have seen more than 20 cases of calcinosis, of which 75 % (12/16) have more than 20 years of experience. At time of JDM diagnosis, 58 % use only history and/or physical exam to screen for calcinosis, 23 % also use imaging and 8 % do no formal assessment. Functional impairment and pain (84 %) were the most common reasons to target calcinosis independent of other treatment for JDM. Increasing systemic immune suppression was first line therapy against calcinosis for 67 % (73/109). 24 % believe a surgeon should evaluate every case of calcinosis if phenotype is amenable to removal. To treat calcinosis, the most frequently used immunomodulator was IVIG (56 %), which was also ranked most successful by 29 % (24/83). Bisphosphonates were the most common (72 %) and ranked most successful (57 %) alternative therapy for calcinosis. IV Sodium Thiosulfate was only used by 9 % (10/106), but was ranked most successful by 30 % (3/10) of those who used it.

**Conclusions**

Calcinosis is a rare but morbid complication of JDM, and experience treating calcinosis is low but varies among physicians. Screening methods for calcinosis and the perceived relationship to active JDM also vary, reflecting low rates of targeted calcinosis therapy separate from overall disease treatment. IVIG and bisphosphonates are most frequently used and considered most successful when treating calcinosis, but many other immunosuppressive and alternative agents are utilized.

**Acknowledgements**

1. Research reported in this publication was supported by the Washington University Institute of Clinical and Translational Sciences grant UL1TR000448 from the National Center for Advancing Translational Sciences (NCATS) of the National Institutes of Health (NIH). The content is solely the responsibility of the authors and does not necessarily represent the official view of the NIH.

PI: Bradley Evanoff, MD, MPH

Project Title: Washington University Institute of Clinical and Translational Sciences

2. Study data were collected and managed using REDCap electronic data capture tools hosted at Washington University in St. Louis.^1^ REDCap (Research Electronic Data Capture) is a secure, web-based application designed to support data capture for research studies, providing 1) an intuitive interface for validated data entry; 2) audit trails for tracking data manipulation and export procedures; 3) automated export procedures for seamless data downloads to common statistical packages; and 4) procedures for importing data from external sources.

^1^Paul A. Harris, Robert Taylor, Robert Thielke, Jonathon Payne, Nathaniel Gonzalez, Jose G. Conde, Research electronic data capture (REDCap) - A metadata-driven methodology and workflow process for providing translational research informatics support, J Biomed Inform. 2009 Apr;42(2):377-81.

## P23 CARRA dermatomyositis CTP pilot study

### Ann Reed^1^, Adam Huber^2^, George Tomlinson^3^, Eleanor Pullenayegum^3^, John Matelski^3^, Y. Ingrid Goh^3^, Laura Schanberg^1^, Brian M. Feldman^3^

#### ^1^Duke University, Durham, NC, USA; ^2^Dalhousie University, Halifax, NS, Canada; ^3^University of Toronto, Toronto, CA

##### **Correspondence:** Brian M. Feldman (brian.feldman@sickkids.ca) – University of Toronto, Toronto, CA

**Background**

We compared 3 consensus treatment strategies in a prospective cohort of children with dermatomyositis (JDM). The 3 strategies were: 1) MP – oral prednisone (pred) and subcutaneous methotrexate (MTX), 2) MMP – intravenous pulse methylpredisolone (IVMP) followed by pred and MTX, and 3) MMPI – intravenous immunuglobulin (IVIG) along with IVMP, MTX and pred. Visits occurred at baseline, 1, 2, 6, 12 and 18 months.

**Results**

The primary outcome was the feasibility of enrolling and following subjects. Exploratory efficacy outcomes included physician global scale (PhysGlob) at 2 months, and manual muscle testing [MMT], childhood myositis scale [CMAS], and steroid dose at 2, 6, 12, and 18 months). Data were analyzed with models that corrected for confounding by indication using Bayesian methods.

Twelve sites enrolled between 1 and 6 subjects each (total n = 39). 27 subjects were white, 6 black, 2 Asian, 3 mixed race and 1 unknown. There were 25 girls and 14 boys. 13 subjects (33 %) were treated with MP, 18 (46 %) with MMP, and 8 (21 %) with MMPI. After correcting for confounding by indication using a multinomial propensity score, the expected CMAS (mean and 95 % credible interval) at 6 months was: MP 48.7 (46.0, 52.4), MMP 48.0 (45.2, 50.6), MMPI 45.1 (41.0, 50.0). The probability that MP results in better CMAS scores at 6 months than MMP is 63 %, and the probability that MP is better than MMPI is 90 %.

While these effectiveness estimates may serve in planning future trials, it is possible that residual confounding by indication might explain the differences we saw.

**Conclusions**

We conclude that it is feasible to use the CARRA CTP method to study comparative effectiveness of treatments for children with JDM.

## P24 Unexpectedly high incidences and prolonged disease activity in children with chronic non-bacterial osteomyelitis (CNO) as compared to bacterial osteomyelitis

### Anja Schnabel^1^, Ursula Range^2^, Gabriele Hahn^3^, Timo Siepmann^4^, Reinhard Berner^1^, and Christian Michael Hedrich^1^

#### ^1^Pediatric Rheumatology and Immunology, Children’s Hospital Dresden, Universitätsklinikum Carl Gustav Carus, Technische Universität Dresden, Dresden, Germany; ^2^Institute for Medical Informatics and Biometry, Universitätsklinikum Carl Gustav Carus, Technische Universität Dresden, Dresden, Germany; ^3^Department of Radiology, Universitätsklinikum Carl Gustav Carus, Technische Universität Dresden, Dresden, Germany; ^4^Center for Clinical Research and Management Education, Division of Health Care Sciences, Dresden International University, Dresden, Germany

##### **Correspondence:** (christian.hedrich@uniklinikum-dresden.de) – Pediatric Rheumatology and Immunology, Children’s Hospital Dresden, Universitätsklinikum Carl Gustav Carus, Technische Universität Dresden, Germany

**Background**

Historically, osteomyelitis was considered an infectious disorder. More recently, inflammatory mechanisms were recognized causing a significant proportion of pediatric osteomyelitis. This study was to compare demographics, presentation, treatment and outcomes in children with chronic non-bacterial (CNO) or bacterial osteomyelitis (BOM).

**Methods**

A chart review of osteomyelitis patients from the departments of pediatrics, pediatric surgery, orthopedic surgery, and oral and maxillofacial surgery was conducted in a tertiary referral center, covering the years 2004-2014.

**Results**

Incidences of CNO (n = 49) and BOM (n = 56) were comparable. Differentiation between CNO and BOM based on clinical or laboratory findings was mostly impossible. However, children with BOM more frequently presented with local inflammatory signs (47 % vs. 68 %, p = 0.040), fever (12 % vs. 38 %, p = 0.003) and abscesses (0 % vs. 39 %, p < 0.001). Peripheral arthritis (14 % vs. 0 %, p < 0.001), bowel disease (10 % vs. 2 %, p = ns) and hyperostosis (29 % vs. 4 %, p = 0.001) were more common in CNO. Whole body MRI was performed in 76 % of CNO patients, unveiling multifocal lesions in 80 % (CRMO). CNO treatment included NSAIDs, prednisolone, TNF-a inhibitors, and bisphosphonates. Over 50 % of CNO patients relapsed after a median of 30 months, while only 42 % sustained clinical remission after 5 years.

**Conclusions**

Though considered a rare disorder, institutional incidences of CNO were comparable to BOM. This is of special importance, since long-term outcomes in CNO were less favorable than previously suspected. Particularly CRMO patients are at risk to develop arthritis and sacroiliitis. Whole body MRIs should be performed in suspected CNO, since clinically inapparent lesions are not uncommon. Prospective studies are warranted to establish evidence-based diagnostic and therapeutic approaches to CNO.

## P25 Juvenile systemic sclerosis cohort within the Childhood Arthritis and Rheumatology Research Alliance (CARRA) Legacy Registry: Follow up characteristics

### Brandi Stevens^1^, Kathryn S. Torok^1^, Suzanne Li^2^, Nicole Hershey^1^, Megan Curran^3^, Gloria Higgins^4^, Katharine Moore^5^, Egla Rabinovich^6^, Anne M. Stevens^7^, for the CARRA Registry Investigators^8^

#### ^1^Children’s Hospital of Pittsburgh of UPMC, Pittsburgh, PA, USA; ^2^Hackensack University Medical Center, Hackensack, NJ, USA; ^3^Northwestern University Feinberg School of Medicine, Chicago, IL, USA; ^4^Nationwide Children's Hospital, Columbus, OH, USA; ^5^Children’s Hospital of Denver, Denver, CO, USA; ^6^ Duke University Medical Center, Durham, NC, USA; ^7^Seattle Children's Research Institute, University of Washington, Seattle, WA, USA; ^8^Childhood Arthritis and Rheumatology Research Alliance, Durham, NC, USA

##### **Correspondence:** Brandi Stevens (brandi.stevens@chp.edu) – Children’s Hospital of Pittsburgh of UPMC, Pittsburgh, PA, USA

**Background**

Systemic sclerosis (SSc) is a rare multisystem autoimmune disease characterized by vasculopathy and organ fibrosis. From 2010-2013, the North American CARRA Legacy Registry enrolled and prospectively followed children with rheumatologic conditions including juvenile systemic sclerosis (jSSc). Objectives were to describe disease manifestations, physician metrics, and patient quality of life (QOL) at enrollment and follow up of jSSc subjects.

**Methods**

Descriptive statistics were used for demographic and clinical features. Mann-Whitney U, Wilcoxon Signed Rank, Fisher’s exact, and McNemar’s tests were used when appropriate for unpaired and paired group comparisons.

**Results**

For the 64 children with SSc in the database, the majority were female (84 %) and Caucasian (78 %); 86 % identified as non-Hispanic. Median age of onset was 10.3 yr while age at first pediatric rheumatology (PRH) evaluation was 11.8 yr, with 23 % having a ≥2 yr delay to PRH. Baseline visit occurred a median of 3.6 yrs after disease onset. Forty-one children (64 %) had ≥ 1 follow up visit recorded, and of those, 25 had a visit between 1 to 2 years after enrollment. For the cumulative 50.7 person years of follow up, there were no mortalities or development of solid organ manifestations (cardiac, pulmonary, renal) after baseline visit. Based on available data, analysis of change in disease over time was based on the 25 subjects with follow up visits within 1-2 yrs after enrollment (median 1.4, IQR 1.05-1.88). To assess for bias, baseline data for this group was compared to the remainder of the cohort (n = 39) with no significant differences in demographics (gender, race, ethnicity) or time to care. Age of disease onset was younger in our follow up subgroup, median 8.9 (IQR 5.1-11.8) years old versus 11.1 (IQR 8.1-14.3) years for those without follow up (*p* = 0.027), which was similarly reflected in age at baseline visit (median 13.4 versus 16.1, *p* = 0.03). Comparison of the baseline data for the entire cohort (n = 64) to follow up data at 1-2 years (n = 25) is presented. Disease manifestations remained generally stable at follow up between these two groups. Paired analysis of baseline and follow up data for the n = 25 subgroup revealed trends of decreased frequency of arthritis (4 to 1) and increased joint contractures (7 to 11), decreased disability by ACR Functional Class (44 % to 25 %), and improved median Physician Global Disease Activity Score from 3 to 2.

**Conclusions**

While younger, our follow up subgroup was otherwise representative of our total cohort. Most organ manifestations were stable with time and for the duration of the study observation period, there was no interval development of major organ involvement or mortality. While limited by the cross sectional nature of enrollment, trends of improvement in disability and disease activity were appreciated after 1-2 years of follow up.

## P26 Development and usability testing of an iPad and desktop psycho-educational game for children with Juvenile Idiopathic Arthritis and their parents

### Jennifer Stinson^1^, Mark Connelly^13^, Adam Huber^2^, Nadia Luca^9^, Lynn Spiegel^1^, Argerie Tsimicalis^3^, Stephanie Luca^1^, Naweed Tajuddin^1^, Roberta Berard^4^, Julia Barsalou^5^, Sarah Campillo^6^, Paul Dancey^7^, Ciaran Duffy^8^, Brian Feldman^1^, Nicole Johnson ^9^, Patrick McGrath^2^, Natalie Shiff^10^, Shirley Tse^1^, Lori Tucker^11^, Charles Victor^12^

#### ^1^The Hospital for Sick Children, University of Toronto, Toronto, Canada; ^2^IWK Health Centre, Halifax, Nova Scotia, Canada; ^3^McGill University, Montreal, Canada; ^4^Children's Hospital of Western Ontario, Canada; ^5^CHU Ste-Justine, Montreal, Quebec, Canada; ^6^Montreal Children's Hospital, Montreal, Canada; ^7^Memorial University of Newfoundland, Newfoundland, Canada; ^8^Children's Hospital of Eastern Ontario, Canada; ^9^Alberta Children's Hospital, Calgary, Alberta, Canada; ^10^University of Florida, Gainesville, FL, USA; ^11^British Columbia Children's Hospital, Vancouver, British Columbia, Canada; ^12^University of Toronto, Toronto, Canada; ^13^University of Kansas Medical Center, Kansas City, MO, USA

##### **Correspondence:** Jennifer Stinson (jennifer.stinson@sickkids.ca) – The Hospital for Sick Children, University of Toronto, Canada

**Background**

Juvenile Idiopathic Arthritis (JIA) is a common chronic childhood illness that causes inflamed and painful joints and can negatively impact health-related quality of life (HRQL). Engaging children in their treatment early on is important so that they are better prepared to more independently manage the disease as they become adolescents and young adults. While educational and self-management programs exist for adolescents and adults with JIA, there are no such programs for younger children. The aim of this study was to develop and assess the usability of a bilingual (English and French) interactive psycho-educational video game for 8 to 11 year old children with JIA. The game modules played within 8-weeks on an iPad or desktop computer aims to help children and parents learn disease management and develop coping skills (e.g., dealing with physical symptoms and feelings of stress or strong emotions) using gamification mechanics.

**Methods**

The core game concept in which players battle physical (i.e., pain, stiffness, and fatigue) symptoms and psychological (e.g., anger, frustration) symptoms associated with JIA was developed in collaboration with stakeholders. Five cycles of game usability testing have been conducted with 19 English-speaking children (average age = 9.9 years; 12 female, 7 male) and 13 parents. One cycle of French testing has been conducted (average age = 10 years, 1 male, 1 female, 2 parents). Participant interaction with the game proceeded in a step-wise manner, with errors and efficiencies documented. Feedback from participants was also provided through a semi-structured interview and these data were analyzed using content analyses.

**Results**

Overall, the response to the game has been positive. Minor suggestions include changing specific graphics, adding more animation, and reducing the amount of text, with no changes to core game mechanics. Some minor navigation errors (e.g., failure to locate functions or follow the recommended screen flow) and presentation errors (e.g., failure to correctly act upon desired information) were documented. Based on participant feedback, English errors and efficiencies were resolved after 5 cycles of testing. French usability testing is ongoing.

**Conclusions**

There may be benefits from educational games for children with JIA with regards to learning self-management strategies in order to improve pain and HRQL. Based upon user suggestions, prototype changes have been implemented and will continue to be tested. The game has been translated into French and will be tested with French-speaking participants. A pilot randomized control trial will assess the feasibility and effectiveness of the game.

## P27 *iCanCope*^TM^: User-centred design and development of a smartphone app to support self-management for youth with arthritis pain

### Jennifer Stinson^1^, Chitra Lalloo^1^, Lauren Harris^1,2,4^, Joseph Cafazzo^3^, Lynn Spiegel^1^, Brian Feldman^1^, Nadia Luca^5^, Ronald Laxer^1^

#### ^1^The Hospital for Sick Children, Toronto, Canada; ^2^University of Toronto, Toronto, Canada; ^3^University Health Network, Toronto, Canada; ^4^Centre for Global eHealth Innovation, Toronto, Canada; ^5^Alberta Children's Hospital, Calgary, Alberta, Canada

##### **Correspondence:** Lauren Harris (lauren.harris@sickkids.ca) – The Hospital for Sick Children, Toronto, Canada

**Background**

Juvenile idiopathic arthritis (JIA) is the most frequent cause of chronic musculoskeletal pain in youth, and can negatively impact all aspects of health-related quality of life. Self-management interventions that provide individuals with disease-specific knowledge, strategies to manage pain symptoms (e.g., cognitive-behavioural therapies or CBT), and social support are needed to improve pain and promote optimal health outcomes for adolescents with JIA. The aim of this research is to design and develop a smartphone-based pain self-management program, called *iCanCope*^TM^*,* for youth living with persistent pain related to JIA.

**Methods**

The *iCanCope*^TM^ program is being developed using a phased approach. In phase 1, we conducted a two-day consensus meeting where experts in pediatric rheumatology, pain, and software development, as well as patients developed standardized self-care pain treatment algorithms for the app. In phase 2 (focus of this abstract), we completed consultations with knowledge users and healthcare providers to iterate the design concepts and features of the app. We then created the iCanCope™ program prototype in collaboration with the Centre for Global eHealth Innovation, including experts in human factors and user experience. The prototype was then refined through iterative consultations with knowledge users, as well as usability testing using semi-structured, audio-recorded individual interviews with youth with JIA.

**Results**

The *iCanCope*™ self-management app is designed to include four key functions: (I) symptom reporting for the areas of pain/inflammation, sleep, mood, physical activity, and energy; (II) personalized goal-setting; (III) coping strategies toolbox including disease education and in-the-moment accessible tools such as meditation; and (IV) social support in the form of a discussion forum which presents scheduled, curated questions for input from the app users. Usability testing was completed in two cycles, in which feedback from knowledge users informed the refinement of functions presented in the first cycle. Specific function refinements included (I) added introductory text for symptom tracking to clarify user expectations; (II) added option for users to select from prepared goals in addition to creating their own custom goals; (III) customized the types of coping strategies that are presented based on user input; and (IV): restructured the social support feature to differentiate from other existing social media platforms and provide unique value. This second iteration of the prototype was validated through usability testing by adolescents with JIA (n = 2). End-users reported that this prototype was easy to use and intuitive to navigate.

**Conclusions**

Smartphone technology has the potential to deliver tailored self-management programming to youth with persistent pain due to JIA. The next phase of this research will be to evaluate the feasibility of the *iCanCope*™ app through a pilot randomized controlled trial with adolescents with JIA across two pediatric rheumatology sites. If found to be effective, the *iCanCope*™ self-management platform will be adapted for other painful conditions and populations.

## P28 Accessing pediatric rheumatology care: Despite barriers, few parents prefer telemedicine

### Danielle R. Bullock^1^, Richard K. Vehe^1^, Lei Zhang^2^, Colleen K. Correll^1^

#### ^1^Division of Pediatric Rheumatology, Department of Pediatrics; University of Minnesota Masonic Children’s Hospital, Minneapolis, MN, USA; ^2^Clinical and Translational Sciences Institute, University of Minnesota; Minneapolis, MN, USA

##### **Correspondence:** Danielle R. Bullock (brue0190@umn.edu) – Division of Pediatric Rheumatology, Department of Pediatrics; University of Minnesota Masonic Children’s Hospital, Minneapolis, MN, USA

**Background**

The United States pediatric rheumatology (PR) workforce is committed to a mission of providing children access to PR care [1]. With a limited number and distribution of pediatric rheumatologists, telemedicine has been proposed as a way to meet this mission [1]. The purpose of this study was to assess interest in telemedicine and to determine which factors affect opinions about this modality among parents/guardians of PR patients in the Upper Midwest.

**Methods**

For six weeks in 2015, English-speaking guardians of patients being evaluated at the University of Minnesota Pediatric Rheumatology Clinic were eligible to participate in a needs-assessment survey. Responses were analyzed using descriptive statistics.

**Results**

Of 221 participants eligible for the survey, 159 (72 %) responded. The majority were guardians of adolescent Caucasian patients and had private insurance. The most common diagnosis was juvenile idiopathic arthritis, and 28 % (45/159) traveled more than three hours to the clinic. An overwhelming majority of respondents (95 %, 144/152) reported a preference for in-person visits over the option of telemedicine. Among 65 respondents who reported travel to the clinic as inconvenient, 92 % still preferred in-person visits. Compared to those for whom travel was not inconvenient, those who indicated travel as inconvenient reported significantly greater difficulty with several barriers to accessing PR care – travel time/distance (p < 0.0001), need for lodging/housing (p = 0.0047), driving in the metro (p < 0.0001), indirect costs (p < 0.0001), parent missing work (p < 0.0001), child missing school (p = 0.0008), and arranging care for other children (p = 0.0219). Nonetheless, preference for in-person visits was similar for both groups (convenient 97 %, inconvenient 92 %; p = 0.2881). Only 8 % (13/158) reported that they or a family member or friend had ever used telemedicine, and this group was more likely to report a preference for telemedicine (27 % vs 3 %; p = 0.0087). This group was also more likely to report telemedicine visits as better than or equal to in-person visits (42 % vs 8 %; p = 0.0033). Neither preference for in-person visits nor views on telemedicine visits compared to in-person visits significantly differed by patient demographics, insurance type, length of time the child was a patient in the clinic, travel time, or frequency of internet use.

**Conclusions**

Despite inconvenient travel and associated barriers, few respondents preferred the option of telemedicine over in-person visits. Familiarity with telemedicine was overall low but positively influenced both a willingness to participate in and the assessment of the quality of telemedicine. Sample bias existed because only those who came to the PR clinic were surveyed. Efforts to increase familiarity with telemedicine may foster increased acceptability. Additionally, other care modalities such as outreach clinics, adult rheumatology care, and shared-care should be explored as means to improve access to PR care.

## P29 Exploration of factors contributing to time to achieve clinically inactive disease (CID) in juvenile idiopathic arthritis (JIA): A preliminary report

### Suhas Ganguli^1^, Max Shenberger^1^, Ritesh Korumilli^2^, Beth Gottlieb^1^

#### ^1^Pediatric Rheumatology, Cohen Children's Medical Center, New York, NY, 11040, USA; ^2^Pediatrics, Flushing Hospital Medical Center, New York, NY, 11355, USA

##### **Correspondence:** Suhas Ganguli (suhaskganguli@gmail.com) – Pediatric Rheumatology, Cohen Children's Medical Center, New York, NY, 11040, USA

**Background**

CID is an important outcome measure for children with JIA. Several demographic, phenotypic and serological factors have been associated with aggressive disease. Early treatment with disease modifying anti-rheumatic drugs (DMARD) and biologic agents have led to favorable outcomes in arthritis in both adults and children. However, the relationship of these factors or early treatment with time to achieve CID has not been well studied in children.

**Objective**

To determine the impact of demographic, phenotypic, serologic and therapeutic factors on the time required to achieve CID in children with JIA.

**Methods**

Demographic, disease subtype (using ILAR definitions), number of active joints, medication use, lab tests for JIA patients enrolled in PR-COIN (Pediatric Rheumatology Care and Outcomes Improvement Network), a multicenter quality improvement collaborative between 1/2011 and 9/2015 were analyzed. Data was analyzed in Microsoft Excel, using t test, correlation, regression and ANOVA. Statistical significance was defined as p < 0.05.

**Results**

Of 272 patients 197 (72.4 %) achieved CID. Oligoarticular extended (OJIA-E) subtype was associated with highest CID (88.8 %) and rheumatoid factor positive (RF+) polyarticular JIA (POJIA) was associated with lowest CID (35.2 %) rates. There was no statistically significant difference among disease subtypes in time required to achieve CID (p = 4.48). Mean duration (t in months) to CID was comparable (p = 1.63) in males (n = 48, t = 55.3) and females (n = 149, t = 65.1). There was no statistically significant difference in time to CID in ANA positive and negative patients with oligoarticular JIA. Time to start DMARD from diagnosis showed positive correlation with time to CID in all subtypes of JIA, with strongest correlation (r^2^ = 0.94) in systemic JIA (SJIA) and enthesitis-related arthritis subgroups (r^2^ = 0.99). Time to start biologic agents showed positive correlation with time to achieve CID in OJIA-E (r^2^ = 0.92), RF negative POJIA (r^2^ = 0.87) and SJIA (r^2^ = 0.58).

**Conclusions**

RF+ POJIA was associated with aggressive disease and with least CID achievement. Gender, disease phenotype or ANA serology were non-contributory to time to achieve CID. Early treatment with DMARD and biologic agents led to faster achievement of CID in most disease subtypes. Analysis of a database with larger sample size will be performed next to confirm these findings.

## P30 Pediatric rheumatology referral patterns: Presenting complaints of new patients at a large, urban academic center

### Martha Rodriguez, Deirdre de Ranieri, Karen Onel, Linda Wagner-Weiner, Melissa Tesher

#### University of Chicago Medicine Comer Children's Hospital, Chicago, IL, USA

##### **Correspondence:** Martha Rodriguez (martha.rodriguez@uchospitals.edu) – University of Chicago Medicine Comer Children's Hospital, Chicago, IL, USA

**Background**

Fewer than 300 certified pediatric rheumatologists currently practice in the United States. This shortage leads to lengthy waits for initial appointments, often resulting in diagnostic delays. On the other hand, many new patient visits are taken by children referred for non-inflammatory musculoskeletal pain or nonspecific laboratory findings. The pediatric rheumatology community has devoted much effort to developing educational programs for pediatric trainees and general pediatricians, but the efficacy of these programs has not been fully assessed.

**Objective**

We aim to assess referral patterns to pediatric rheumatology, via a review of chief complaints and diagnoses for new pediatric rheumatology patients.

**Methods**

A retrospective study was performed of 151 new patient visits from January to May 2015 to the pediatric rheumatology outpatient clinic at Comer Children's Hospital of the University of Chicago. Data were collected on patient sex, age, chief complaint and whether rheumatology follow-up was recommended.

**Results**

Most new patients (60 %) did not have a confirmed rheumatologic diagnosis, and 40 % were requested to return for further rheumatologic follow-up. Female patients were slightly more likely to require rheumatologic follow up (44 %) vs male patients (32 %). The most common presenting complaint was musculoskeletal pain, 41 %, followed by abnormal labs, 33 %. Rheumatology follow-up was recommended for only 22 % of patients presenting with musculoskeletal pain, and 33 % of those with abnormal lab testing. Joint swelling and fever were most predictive of need further rheumatologic evaluation (83 % and 86 % respectively).

**Conclusions**

At our center, musculoskeletal pain was the most common chief complaint. Patients presenting with musculoskeletal pain were unlikely to have a rheumatologic disease. These findings are consistent with previous studies. Joint swelling and fever were most likely to be associated with need for further rheumatologic evaluation. Encouragingly, in comparison with earlier investigations, a smaller proportion of non-rheumatologic patients were referred to the pediatric rheumatology clinic. However, a knowledge gap persists among referring physicians, particularly with regard to the assessment of rheumatologic vs non-rheumatologic musculoskeletal pain in children.

## P31 Quality improvement (QI) initiatives in childhood systemic lupus erythematosus (cSLE)

### Elizabeth Roth Wojcicki^1^, Kristyn L. Maletta^2^, Dominic O. Co^1^, Marsha Malloy^1^, Sarah Thomson^3^, Judyann C. Olson^1^

#### ^1^Medical College of WI, Milwaukee, WI, USA; ^2^National Outcomes Center, Milwaukee, WI, USA; ^3^Children's Hospital of Wisconsin, Milwaukee, WI, USA

##### **Correspondence:** Elizabeth Roth Wojcicki (eroth@mcw.edu) – Medical College of WI, Milwaukee, WI, USA

**Background**

Children represent about 15-20 % of the SLE population and accrue a higher disease burden over a longer time. Consensus QI indicators for cSLE were published in 2013. (1) Our recent work has targeted 2 of these: vaccination against influenza, pneumococcus, meningococcus, and haemophilus influenza (Hib) and annual eye screening. We report the rates at which our cSLE patients are up to date on eye exams, immunizations, and documentation of patient education related to these goals.

**Methods**

All patients met the ACR 1982/1997 Revised Criteria for Classification of SLE and were diagnosed before age 18. We used immunization data from the Wisconsin Immunization Registry. Data for eye exams was obtained manually. Previous work created a pre visit planning (PVP) process to identify educational topics that each patient needed and pertinent teaching materials were given. A SLE specific flowsheet was implemented in our electronic medical record to document education. In this cycle of QI, the flowsheet was used to determine what education was needed. In addition, we continued our system of immunization reminders for pneumococcus and transferred this to include seasonal influenza. Finally, we established the baseline immunization rates for Hib, meningococcus and influenza.

**Conclusions**

Using both simple interventions and modifications of our electronic medical record (EMR), we were able to standardize the care of our cSLE patients by systematically providing periodic education regarding the importance of immunizations, eye screening and reproductive health. We improved rates pneumococcal immunization and established baseline immunization rates for Hib, meningococcal and influenza immunization that will allow us to track our progress in improving their rates of administration. Interestingly, rates for performance for all measures were better than the rates for documentation of education. This was most striking for Hib for which education rate was 24.5 %, but the immunization rate was 86.8 %. The high immunization rate for Hib, meningococcal and influenza vaccines reflects administration in the primary care clinic in addition to the rheumatology clinic, and if a provider sees that a vaccine was performed, they are unlikely to perform re-education. In future cycles of quality improvement, we plan to build on this success to improve adherence to eye screening recommendations and provision of reproductive health services to our cSLE patients. We plan to leverage our EMR’s data collection and report functions to make the process more efficient. This will allow us to take on more quality improvement goals with the time and resources we currently have available.

## P32 Proliferative lupus nephritis in juvenile SLE: Support from the pediatric nephrology community for the definitions of responsiveness and flare in the 2012 consensus treatment plans

### Scott E. Wenderfer^1^, Mileka Gilbert ^2^, Joyce Hsu^3^, Sangeeta Sule^4^, Tamar B. Rubinstein^5^, Beatrice Goilav^5^, Daryl M. Okamura^6^, Annabelle Chua^7^, Laurence A. Greenbaum^8^, Jerome C. Lane^9^, Emily von Scheven^10^, Stacy P. Ardoin^11^ and Natasha M. Ruth^2^

#### ^1^Baylor College of Medicine, Houston, TX, USA; ^2^Medical University of South Carolina, Charleston, SC, USA; ^3^Stanford University, Stanford, CA, USA; ^4^Johns Hopkins Children’s Hospital, Baltimore, MD, USA; ^5^Children’s Hospital at Montefiore, Bronx, NY, USA; ^6^Seattle Children’s Hospital, Seattle, WA, USA; ^7^Duke University, Durham, NC, USA; ^8^Emory University, Atlanta, GA, USA; ^9^Northwestern University, Evanston, IL, USA; ^10^University of California San Francisco, San Francisco, CA, USA; ^11^Nationwide Children’s Hospital, Columbus, OH, USA

##### **Correspondence:** Scott E. Wenderfer (wenderfe@bcm.edu) – Baylor College of Medicine, Houston, TX, USA

**Background**

Consensus treatment plans (CTPs) for induction therapy of newly diagnosed proliferative lupus nephritis in juvenile SLE were published by CARRA in March 2012 (Mina *et al.*, Arthritis Care & Research). For defining outcome measures (nephritis responsiveness and flare) in these CTPs, pediatric rheumatologists selected the definitions from the American College of Rheumatology (Arthritis Rheum. 2006) and the European League against Rheumatism (Gordon *et al*., Lupus 2009). These definitions were originally developed for the study of proliferative nephritis in adult SLE.

**Methods**

The objective of this study was to assess the level of agreement with the definitions of nephritis responsiveness and flare among pediatric nephrologists. A web- based survey was distributed to the membership of the American Society for Pediatric Nephrology (ASPN) in November 2015.

**Results**

79 pediatric nephrologists completed all survey questions, which represents 15 % of practicing pediatric nephrologists in the United States. Respondents practice at both small (33 %) and large programs (49 %, including 18 % from sites following >100 active SLE patients) in all geographic regions. The number of years since completion of fellowship was evenly distributed (8 % were in practice <2 yrs; 21 %, 2-5 yrs; 21 %, 5- 10 yrs; and 49 %, >10 yrs). Respondents tended to either “strongly agree” or “somewhat agree” with the CARRA definition for each level of responsiveness to therapy. However, nephrologists were most likely to “strongly agree” with the definition of substantial response or no response vs. “somewhat agree” for intermediate responses. Only ~1/3 of respondents “strongly agreed” with the CARRA definitions of proteinuric or nephritic flares; “somewhat agree” was the most common response.

**Conclusions**

A general consensus exists between pediatric rheumatologists and nephrologists in the definitions of response and flare in children being treated for proliferative lupus nephritis. However, there are gaps in agreement that may be strengthened by the use of multi-disciplinary teams in the development of future CTPs. A consensus multi-disciplinary approach to managing these patients may result in improved outcomes in pediatric SLE.

## P33 The steroid taper app: Making of a mobile app

### Jennifer M. P. Woo^1^, Marsha M. Malloy^2^, James A. Jegers^4^, Dustin J. Hahn^1^, Mary K. Hintermeyer^3^, Stacey M. Martinetti^2^, Gretchen R. Heckel^3^, Elizabeth L. Roth-Wojcicki^2^, Dominic O. Co^2^

#### ^1^University of Wisconsin-Milwaukee, Milwaukee, WI, USA; ^2^Medical College of Wisconsin, Milwaukee, WI, USA; ^3^Children’s Hospital of Wisconsin, Milwaukee, WI, USA; ^4^Parent of patient, Milwaukee, WI, USA

##### **Correspondence:** Dominic O. Co (dco@mcw.edu) – Medical College of Wisconsin, Milwaukee, WI, USA

**Background**

Long-term steroid regimens must be tapered gradually to prevent adrenal insufficiency and/or flares of the underlying inflammatory disease. These frequent changes in steroid dose can confuse patients and their families, leading to poor patient compliance. Automation of the steroid tapering process could improve efficiency and decrease variability of adherence and taper regimens. As a result, we pursued the development of a mobile app to address these issues and aid physicians in the prescription of steroid tapers.

**Methods**

We surveyed a spectrum of care providers (both adult and pediatric) representing different specialties and disciplines (e.g., nurses, physicians, advanced practice providers, pharmacists) to assess needs. A patient’s father also joined our team for his perspective as a parent and his technical expertise as a software engineer. After assessing available apps, we determined that an app to calculate steroid tapers represented a gap in the market. The collaboration between the Medical College of Wisconsin and student developers from the University of Wisconsin-Milwaukee “App Brewery” resulted in the development of an app for care providers to calculate a taper and to generate a patient-friendly calendar.

**Results**

We first defined the scope of version 1 of the Steroid Taper App and triaged features for later versions. Next, we broke down the process of steroid taper calculation into automatable steps. We then designed the user interface for the provider and output for the patient. We performed 18 development iterations over 22 months, to produce version 1 of the Steroid Taper App. The app will be publicly available for iOS and android devices pending approval from the iTunes and Google Play Stores.

**Conclusions**

Mobile apps have the potential to automate tedious but important tasks to deliver personalized healthcare and improve patient outcomes. However, the app development process can be daunting for those without an information technology background. Here we present our experience developing a mobile app that automates calculation of steroid tapers and generates a patient-friendly calendar that aims to improve compliance with steroid tapers. We learned the value of engaging all stakeholders, which in this case included patients/families and care providers of a wide range of specialties and from across the continuum of care. We also learned the importance of limiting the scope of the initial version of the app while retaining the capacity and versatility to accommodate new features. We hope that sharing our experience will help others who may be interested in developing a mobile app of their own. In future work, we hope to see the app evolve to include features such as consensus taper protocols and more patient-centered features (i.e., integration into patients’ mobile device calendars and automated dose reminders).

